# Downregulation of MUC15 by miR-183-5p.1 promotes liver tumor-initiating cells properties and tumorigenesis via regulating c-MET/PI3K/AKT/SOX2 axis

**DOI:** 10.1038/s41419-022-04652-9

**Published:** 2022-03-02

**Authors:** Tao Han, Hao Zheng, Jin Zhang, Pinghua Yang, Hengyu Li, Zhangjun Cheng, Daimin Xiang, Ruoyu Wang

**Affiliations:** 1 Department of Oncology, The First Affiliated Hospital of China Medical University, 110001 Shenyang, China; 2 Department of Hepatic Surgery, Third Affiliated Hospital of Second Military Medical University, 200438 Shanghai, China; 3 Key Laboratory of Signaling Regulation and Targeting Therapy of Hepatocellular Carcinoma Ministry of Education, 200438 Shanghai, China; 4 Shanghai Key Laboratory of Hepatobiliary Tumor Biology, 200438 Shanghai, China; 5 Department of Reproductive Heredity Center, Changhai Hospital, Second Military Medical University, 200433 Shanghai, People’s Republic of China; 6 Department of General Surgery, Changhai Hospital, Second Military Medical University, 200438 Shanghai, China; 7 Department of Hepato-Pancreato-Biliary Centers, Zhong Da Hospital, School of Medicine, Southeast University, 210009 Nanjing, China; 8 State Key Laboratory of Oncogenes and Related Genes, Shanghai Cancer Institute, Renji Hospital, Shanghai Jiao Tong University School of Medicine, 200127 Shanghai, China

**Keywords:** Cancer stem cells, Tumour biomarkers, Liver cancer

## Abstract

Mucin 15 (MUC15) is reportedly aberrant in human malignancies, including hepatocellular carcinoma (HCC). However, the role of MUC15 in the regulation of liver tumor-initiating cells (T-ICs) remains unknown. Here, we report that expression of MUC15 is downregulated in liver T-ICs, chemoresistance and recurrent HCC samples. Functional studies reveal that MUC15 inhibits hepatoma cells self-renewal, malignant proliferation, tumorigenicity, and chemoresistance. Mechanistically, MUC15 interacts with c-MET and subsequently inactivates the PI3K/AKT/SOX2 signaling pathway. Moreover, we find that miR-183-5p.1 directly targets MUC15 3′-UTR in liver T-ICs. Coincidentally, SOX2 feedback inhibits MUC15 expression by directly transactivating miR-183-5p.1, thus completing a feedforward regulatory circuit in liver T-ICs. Importantly, MUC15/c-MET/PI3K/AKT/SOX2 axis determines the responses of hepatoma cells to lenvatinib treatment, and MUC15 overexpression abrogated lenvatinib resistance. Analysis of patient cohort, patient-derived tumor organoids and patient-derived xenografts further suggests that the MUC15 may predict lenvatinib benefits in HCC patients. Collectively, our findings suggest the crucial role of the miR-183-5p.1/MUC15/c-MET/PI3K/AKT/SOX2 regulatory circuit in regulating liver T-ICs properties, suggesting potential therapeutic targets for HCC.

## Introduction

The incidence of hepatocellular carcinoma (HCC) has been rising throughout the world. Due to the unobvious symptoms of HCC, most HCC patients are diagnosed at advanced stage, and in patients with advanced HCC approximately 80% will develop recurrence after operation [1]. In the past, advanced HCC was a disease with dismal prognosis and few treatment options. Currently, targeted drugs are the first-line treatment for patients with HCC who are not eligible for surgery [2, 3]. Due to the genetic heterogeneity of HCC, only a small proportion of patients respond to targeted drugs, and the majority of patients not only have no curative effect, but also have very serious side effects. Thus, it is urgent to elucidate the underlying mechanisms of drugs resistance and discover reliable biomarkers that can predict drugs response in HCC patients.

Tumor-initiating cells (T-ICs) or cancer stem cells (CSCs) were first identified in hematopoietic tumors and subsequently in solid tumors, such as rectal, breast and brain cancers [4, 5]. T-ICs accounts for a small proportion in tumor tissue cells and have the ability of self-renewal, infinite proliferation and multidirectional differentiation, which plays a key role in the process of tumor initiation, metastasis, chemo-resistance, and recurrence [6, 7]. The introduction of T-ICs not only brings new views for the pathogenesis of tumor development and recurrence, but also provides a new theoretical basis for clinical diagnosis and treatment of tumors. Tumors that harbor an abundant T-IC population may signal a poor clinical outcome in HCC patients [8]. Therefore, in-depth studies on the regulatory mechanism of liver T-ICs and finding appropriate intervention targets are expected to provide new ideas for the treatment of HCC.

Mucin 15 (MUC15) is a membrane-associated mucin that belongs to transmembrane family of mucins, which generally function primarily in the hydration, lubrication, and protection of epithelial surfaces [9]. MUC15 protein consists of highly conserved cytoplasmic tail domain, extracellular domain and small transmembrane domain, suggesting that MUC15 may play an important role in cell signal transduction [10]. MUC15 is reported to either facilitate or impair carcinogenesis in different cancer types. For example, MUC15 plays an oncogenic role in pancreatic, gastric, colon, and thyroid cancer [11–14]. Moreover, our previous study demonstrated that MUC15 downregulation correlated with advanced stage, poor differentiation, and metastasis of liver cancer [15]. We further elucidated that MUC15 suppressed HCC metastasis via inhibiting EGFR dimerization and PI3K-Akt signal. Nevertheless, the regulatory role of MUC15 in liver T-ICs remains unknown.

In this study, we discover that MUC15 was downregulated in liver T-ICs and played an essential role in hepatoma cells self-renewal, malignant proliferation, tumorigenicity, and chemoresistance, suggesting MUC15 is a novel biomarker for liver T-ICs and a potential target for HCC therapy.

## Results

### MUC15 interference can induce hepatocellular malignant transformation

**Fig. 1:**
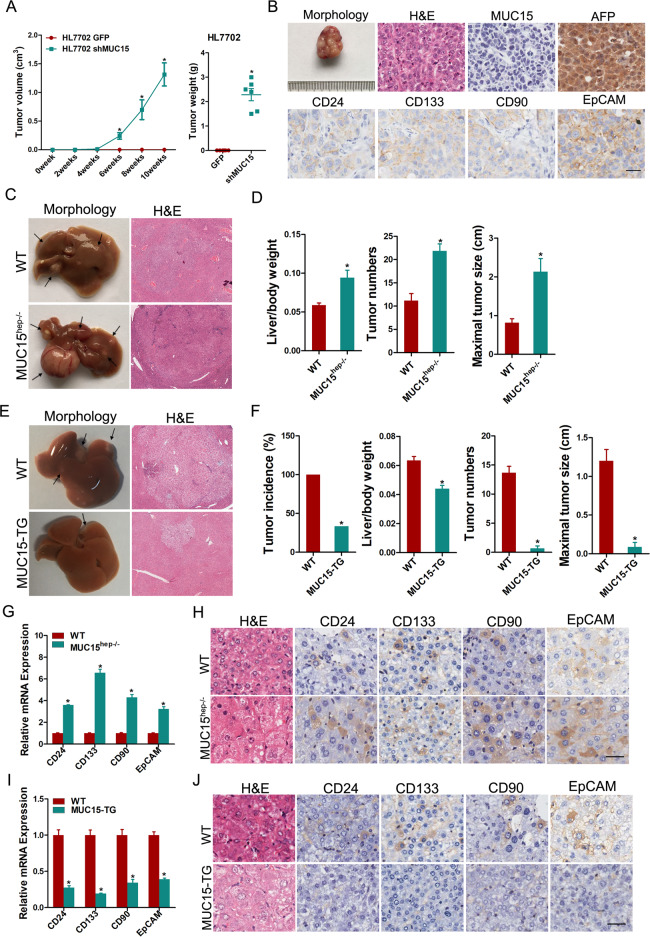
MUC15 suppresses hepatocellular oncogenesis via inhibiting T-ICs generation. **A** HL7702 shMUC15 and control cells were injected subcutaneously into NOD-SCID mice at 1 × 10^3^ cells per mouse. Xenografted tumor growth was monitored, and tumor weight was measured 10 weeks later. **B** The tumor formed in **E** was subjected to H&E and IHC staining. Scale bar = 25 μm. **C** Representative images and H&E staining of MUC15^hep−/−^ and WT mice at 5 months after DEN injection (*n* = 6). **D** The tumor numbers, maximal tumor sizes and liver-to-body weight ratios of MUC15^hep−/−^ and WT mice at 5 months after DEN injection was measured. **E** Representative images and H&E staining of MUC15-TG and WT mice at 5 months after DEN injection (*n* = 6). **F** The tumor incidence, tumor numbers, maximal tumor sizes, and liver-to-body weight ratios of MUC15-TG and WT mice at 5 months after DEN injection was measured. **G**, **H** The mRNA and protein expression of TIC-associated markers in tumors from MUC15^hep−/−^ and WT mice at 5 months after DEN injection were examined by real-time PCR and IHC staining analysis. Scale bar = 25 μm. **I**, **J** The mRNA and protein expression of TIC-associated markers in tumors from MUC15-TG and WT mice at 5 months after DEN injection were examined by real-time PCR and IHC staining analysis. Scale bar = 25 μm. All results are presented as the mean ± SD, and statistical significance was assessed using a two-tailed Student *t*-test. *p < 0.05.

High-grade dysplastic nodules (HGDNs) are regarded as a precancerous lesion for hepatocarcinogenesis, and the majority of human HCCs arise from HGDNs [16]. Immunostaining analysis showed that MUC15 expression was significantly downregulated in seven of ten HGDNs, suggesting that the potential role of MUC15 in cell transformation (Supplementary Fig. S1A). To elucidate the tumor suppressor role of MUC15 in liver, we suppressed MUC15 in normal liver cell lines (HL7702 and THLE3) (Supplementary Fig. S1B, C). Interestingly, MUC15 interference dramatically elevated the expression of T-IC-associated markers in liver cells and facilitated the generation of T-ICs (Supplementary Fig. S1D, E). Moreover, the interference of MUC15 induced hepatocyte transformation in vitro (Supplementary Fig. S1F) and resulted in tumor formation in a murine subcutaneous tumor model while the control cells did not (Fig. 1A). Convincingly, the formed tumor exhibited the phenotype of HCC with strong alpha fetal protein (AFP) staining and T-IC-associated markers (Fig. 1B).

### MUC15 suppresses hepatocellular oncogenesis via inhibiting T-ICs generation

To further elucidated the roles of MUC15 in hepatocarcinogenesis, we generated hepatocyte-specific MUC15 deleted mice and hepatocyte-specific MUC15 transgenic (TG) mice. MUC15 was deleted in hepatocytes in MUC15^hep−/−^ mice that generated by crossing MUC15^flox/flox^ mice with Albumin-Cre transgenic mice (Supplementary Fig. S2A). Hepatocyte-specific MUC15-TG mice were generated by crossing MUC15^loxp/loxp^ mice with Albumin-Cre transgenic mice (Supplementary Fig. S2B). The MUC15 expression in healthy liver was examined by real-time PCR and western blotting. Our data showed that MUC15 was undetectable in MUC15^hep−/−^ mice (Supplementary Fig. S2C). We also observed that increased expression of MUC15 in hepatocytes of transgenic mice compared with wild-type (WT) mice (Supplementary Fig. S2D).

To generate HCC, MUC15^hep−/−^ or MUC15-TG mice and their control mice were treated with DEN to initiate tumor growth, followed by administration of CCL4 twice a week for 2 months to promote tumor growth (Supplementary Fig. S2E). All the male animals in both control and MUC15^hep−/−^ groups developed visible hepatic tumor foci (Fig. 1C). However, hepatocyte-specific deletion of MUC15 dramatically increased the tumor numbers, maximal tumor sizes and liver-to-body weight ratios (Fig. 1D). Conversely, the formation of liver cancer was significantly reduced in MUC15-TG mice compared with that in WT mice (Fig. 1E). Furthermore, the tumor load was markedly reduced by MUC15 overexpression, as indicated by a significant decline in tumor incidence, numbers, maximal tumor sizes and liver-to-body weight ratios in MUC15-TG mice compared with control mice (Fig. 1F). Ki67 staining showed a notable increase in hepatoma cell proliferation in MUC15^hep−/−^ mice and an evidently decrease in hepatoma cell proliferation in MUC15-TG mice compared with their WT mice (Supplementary Fig. S2F, G). There were no differences in the levels of aspartate aminotransferase (AST) and alanine aminotransferase (ALT) between MUC15^hep−/−^ or MUC15-TG and their WT mice (Supplementary Fig. S2H, I), suggesting that MUC15 deletion or overexpressing did not influence the liver injury upon DEN stimuli. In addition, the difference of hepatic macrophage infiltration was less noticeable between MUC15^hep−/−^ or MUC15-TG and their WT mice (Supplementary Fig. S2J, K). Importantly, the expression of TIC-associated markers, including CD24, CD133, CD90, and EpCAM was markedly elevated in HCCs from MUC15-deficient mice and significantly declined in HCCs from MUC15-overexpressing mice (Fig. 1G–J), which further suggesting that MUC15 impairs T-ICs generation and inhibits liver oncogenesis.

### MUC15 inhibits liver T-ICs expansion

**Fig. 2:**
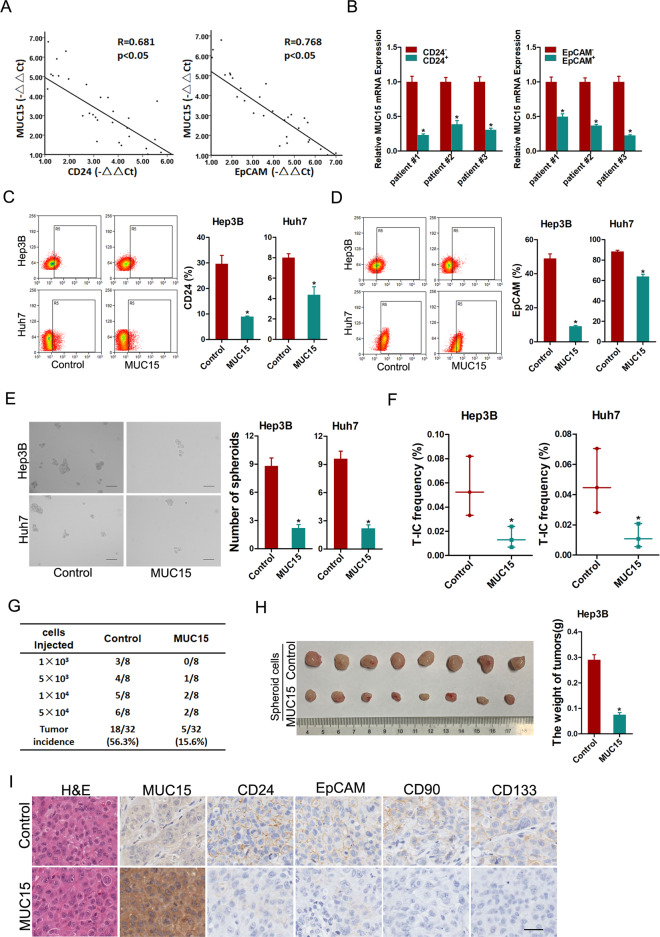
MUC15 inhibits liver T-ICs expansion. **A** The correlation between the level of MUC15 and CD24 (left) or EpCAM (right) in primary HCC cells (*n* = 30) was determined by real-time PCR analysis. **B** Real-time PCR analysis MUC15 expression in sorted CD24^+^ (left) or EpCAM^+^ (right) primary HCC cells relative to negative cells. **C**, **D** Flow cytometry analysis of CD24^+^ or EpCAM^+^ populations in spheroids generated from MUC15 overexpression hepatoma cells and control cells. **E** Representative images of hepatoma spheroids generated from MUC15 overexpression hepatoma cells and control cells. The number of spheroids was counted and compared. **F** The frequency of liver T-ICs in MUC15 overexpression hepatoma cells and control cells was compared by in vitro limiting dilution assay. **G** Hepatoma cells dissociated from Hep3B MUC15 spheroids or control spheroids were inoculated into NOD-SCID mice subcutaneously, and the tumorigenicity was evaluated two months post inoculation. **H** In total, 2 × 10^6^ cells dissociated from Hep3B-MUC15 or Hep3B-control spheroids were subcutaneously inoculated into NOD-SCID mice (*n* = 8) and excised 7 weeks later. The weight of xenografed tumors from different groups was compared. **I** Representative images of immunohistochemical staining of liver T-IC markers in xenografted tumors. Scale bar = 25 μm. All results are presented as the mean ± SD, and statistical significance was assessed using a two-tailed Student *t*-test. *p < 0.05.

CD24 and EpCAM are well-accepted liver T-IC markers [17, 18]. In tumor cells isolated from primary HCC tissues, pearson correlation analysis revealed that MUC15 levels were negatively correlated with the expression of CD24 and EpCAM (Fig. 2A). To determine the expression of MUC15 in liver T-ICs, we enriched T-ICs by flow cytometry sorting or sphere formation. As shown in Fig. 2B, MUC15 levels were downregulated in sorted CD24^+^ or EpCAM^+^ formed primary HCC cells. Notably, MUC15 level was decreased in HCC spheres and recovered to origin level when the spheres were reattached (Supplementary Fig. S3A, B). Two HCC cell lines showed the similar results (Supplementary Fig. S3C–F). Moreover, MUC15 expression was much lower in lenvatinib resistant HCC patient tissues than lenvatinib sensitive HCC patient tissues (Supplementary Fig. S3G). Coincidentally, we observed that MUC15 expression was notably decreased in recurrent HCC compared with the primary lesion (Supplementary Fig. S3H). These data indicated that MUC15 was preferentially downregulated in liver T-ICs.

Next, we found that MUC15 overexpression downregulated the expression of liver T-ICs markers and stemness-associated transcription factors in hepatoma spheroids (Supplementary Fig. S3I–K). Flow cytometry analysis revealed that the population of CD24^+^ or EpCAM^+^ cells in HCC spheroids was decreased by MUC15 overexpression (Fig. 2C, D). Spheroid formation was attenuated in MUC15 overexpression hepatoma cells (Fig. 2E). Furthermore, in vitro limited dilution assays revealed that ectopic MUC15 expression attenuated the TIC-like properties in hepatoma cells (Fig. 2F).

To further determine the effect of MUC15 on the tumorigenicity of liver T-ICs, sphere-derived MUC15 overexpression or control cells were inoculated into NOD/SCID mice. In vivo limiting dilution assay revealed that overexpression of MUC15 significantly reduced tumor incidence and T-IC frequency (Fig. 2G). In further study, we found that hepatoma cells from MUC15 overexpression spheroids exhibited attenuated xenografted tumor growth, tumor size, and tumor weight in vivo (Fig. 2H), suggesting the inhibitory role of MUC15 in liver T-ICs propagation and HCC progression. Consistently, decreased CD24^+^, EpCAM^+^, CD133^+^, and CD90^+^ liver T-ICs were detected in MUC15 overexpression spheroid-formed xenografts compared with control xenografts (Fig. 2I), which further indicates that MUC15 inhibits the expansion of liver T-ICs.

### MUC15 suppresses liver T-ICs expansion via inhibiting PI3K/AKT/SOX2 activation

**Fig. 3:**
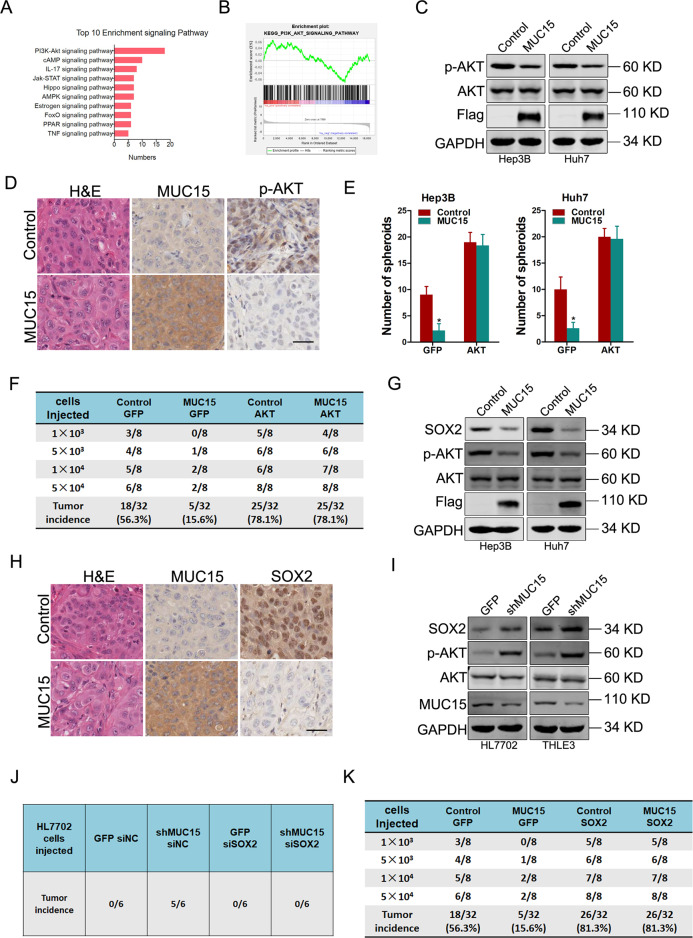
MUC15 suppresses PI3K/AKT/SOX2 activation in liver T-ICs. **A** Pathway enrichment analysis of differentially expressed genes from RNA-seq of sphere-derived MUC15 overexpression or control cells. **B** Gene set enrichment analysis shows the enrichment of gene sets positive related to PI3K/AKT pathway in sphere-derived MUC15 overexpression or control cells. **C** Phosphorylation of AKT in Hep3B/Huh7 MUC15 and control spheroids was determined by western blot. **D** Immunohistochemical staining of p-AKT in xenografted tumors generated by Hep3B MUC15 and control spheroid. Scale bar = 25 μm. **E** Spheres formation assay of Hep3B/Huh7 MUC15 and control cells infected with AKT overexpression virus. The number of spheroids was counted and compared. **F** Hep3B MUC15 and control cells infected with AKT overexpression virus were subjected to in vivo limiting dilution assay. Tumors were observed over 2 months; *n* = 8 for each group. **G** The expression of SOX2 in Hep3B/Huh7 MUC15 and control spheroids was determined by western blot. **H** Immunohistochemical staining of SOX2 in xenografted tumors generated by Hep3B MUC15 and control spheroid. Scale bar = 25 μm. **I** The expression of p-AKT and SOX2 in shMUC15 and control hepatocyte cells was determined by western blot. **J** HL7702 shMUC15 and control cells were transfected with siSOX2 or siNC and were then injected subcutaneously into NOD-SCID mice at 1 × 10^3^ cells per mouse. Xenografted tumor formation was monitored 10 weeks later. **K** In vivo limiting dilution assay of indicated HCC cells. Tumors were observed over 2 months; *n* = 8 for each group. All results are presented as the mean ± SD, and statistical significance was assessed using a two-tailed Student *t*-test. *p < 0.05.

To elucidate the mechanism underlying the suppressive role of MUC15 in liver T-ICs expansion, we profiled gene expression in sphere-derived MUC15 overexpression or control cells using RNA sequencing. Gene ontology (Fig. 3A) and gene set enrichment analysis (Fig. 3B) revealed that MUC15 overexpression resulted in enrichment of gene sets related to PI3K/AKT pathway. Western blot analysis further confirmed that p-AKT was downregulated in MUC15 overexpression hepatoma spheroids (Fig. 3C). Consistently, decreased p-AKT level was detected in MUC15 overexpression spheroids formed xenografts (Fig. 3D). Moreover, Kinase activity assays showed that PI3K was also downregulated by MUC15 overexpression in liver T-ICs (Supplementary Fig. S4A). Furthermore, the self-renewal ability, liver T-ICs frequency and tumorigenesis capacity in MUC15 overexpression hepatoma cells can be restored through introduction of AKT (Fig. 3E, F and Supplementary Fig. S4B).

The transcription factor SOX2 controls tumor initiation and T-ICs functions, and its expression is also regulated via AKT [19]. Notably, western blot analysis found that SOX2 was also downregulated in MUC15 overexpression hepatoma spheroids (Fig. 3G). Consistently, decreased SOX2 level was detected in MUC15 overexpression spheroids formed xenografts (Fig. 3H). Moreover, the expression p-AKT and SOX2 was upregulated in MUC15 knockdown hepatocyte cells (Fig. 3I). Interestingly, treatment with AKT siRNA completely blocked the effects of MUC15 knockdown on SOX2 upregulation (Supplementary Fig. S4C). Restoration of AKT capitulated the expression of SOX2 in MUC15 overexpression HCC spheres (Supplementary Fig. S4D). More importantly, the interference of SOX2 abolished the enhancement of self-renewal, colony formation, and tumorigenesis triggered by MUC15 knockdown in normal hepatocytes (Fig. 3J and Supplementary Fig. S4E, F). While introduction of SOX2 restored the self-renewal ability, liver T-ICs frequency and tumorigenesis capacity in MUC15 overexpression HCC spheres (Fig. 3K and Supplementary Fig. S4G–I).

### MUC15 modulates c-MET/PI3K/AKT/SOX2 signaling in liver T-ICs

**Fig. 4:**
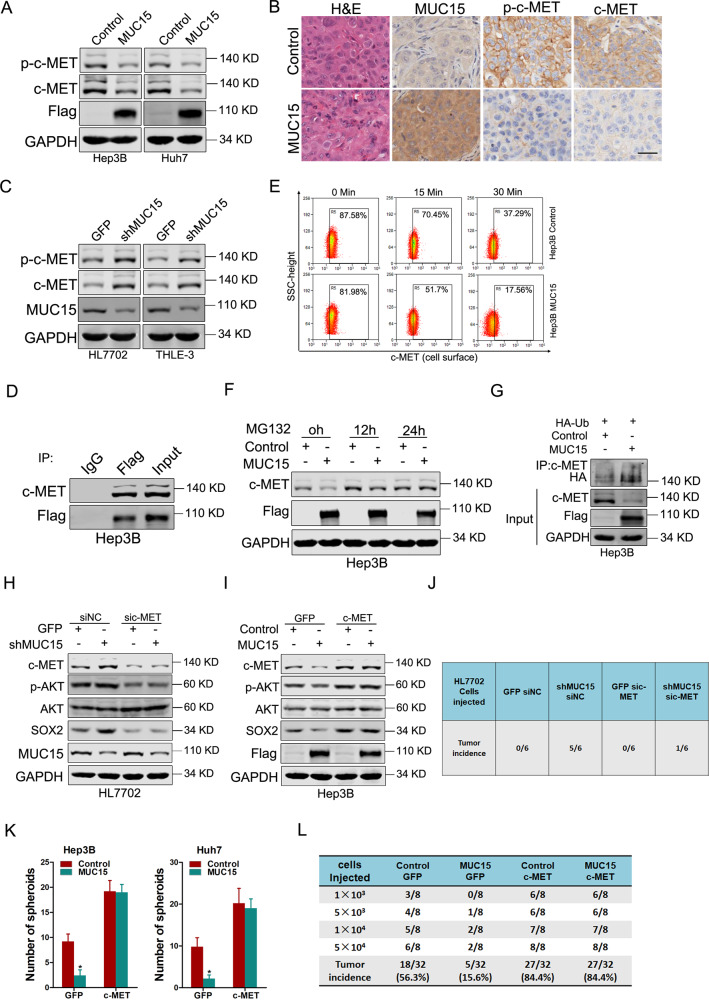
MUC15 modulates c-MET/PI3K/AKT/SOX2 signaling in liver T-ICs. **A** Phosphorylation of c-MET and total c-MET in Hep3B/Huh7 MUC15 and control spheroids was determined by western blot. **B** Immunohistochemical staining of p-c-MET and c-MET in xenografted tumors generated by Hep3B MUC15 and control spheroid. Scale bar = 25 μm. **C** The expression of p-c-MET and c-MET in shMUC15 and control hepatocyte cells was determined by western blot. **D** Endogenous c-MET and Flag-tagged MUC15 were immunoprecipitated. **E** The cell surface expression levels of c-MET in Hep3B MUC15 and control spheroids were determined using flow cytometry. **F** Hep3B MUC15 and control spheroids were treated with MG132 (20 μM) for indicated times and then subjected to western-blot analysis. **G** Hep3B MUC15 and control spheroids were transfected with hemagglutinin (HA)-tagged ubiquitin and then total ubiquitinated proteins were detected by Western blotting for HA. **H** HL7702 shMUC15 and control cells transfected with sic-MET or siNC were subjected to western blot analysis. **I** Hep3B MUC15 and control cells infected with c-MET overexpression virus were subjected to western blot analysis. **J** HL7702 shMUC15 and control cells were transfected with sic-MET or siNC and were then injected subcutaneously into NOD-SCID mice at 1 × 10^3^ cells per mouse. Xenografted tumor formation was monitored 10 weeks later. **K** Hep3B/Huh7 MUC15 and control cells were infected with c-MET overexpression virus and were then subjected to spheroids formation assay. **L** In vivo limiting dilution assay of indicated HCC cells. Tumors were observed over 2 months; *n* = 8 for each group. All results are presented as the mean ± SD, and statistical significance was assessed using a two-tailed Student *t*-test. *p < 0.05.

It has been well accepted that the activation of PI3K/AKT is predominantly regulated by the upstream tyrosine kinases [20]. Herein our data showed that EGFR, FGFR1, VEGFR3, RET, PYK2, or FLT3 was not influenced by MUC15 overexpression (Supplementary Fig. S5A). However, c-MET was reduced in MUC15 overexpression hepatoma spheroids (Fig. 4A). Consistently, decreased c-MET level was observed in MUC15 overexpression spheroids formed xenografts (Fig. 4B). Moreover, the expression c-MET was upregulated in MUC15 knockdown hepatocyte cells (Fig. 4C). By co-immunoprecipitation (coIP) experiments, the interactions between endogenously expressed MUC15 and c-MET were confirmed in Hep3B cells (Fig. 4D). To establish how MUC15 controls c-MET activation, we assessed whether MUC15 regulates c-MET internalization kinetics via FACS analysis of c-MET levels at the surfaces. After HGF stimulation, MUC15-overexpressing cells internalized c-MET faster than control cells (Fig. 4E). Next, we explored the mechanisms about MUC15 regulating c-MET at transcriptional or post-transcriptional levels. As shown in Fig. 4F and Supplementary Fig. S5B, downregulation of c-MET by MUC15 overexpression was blocked by the proteasome inhibitor MG132, but not by inhibitors of protein synthesis cycloheximide (CHX), implying that MUC15 modulates c-MET protein degradation rather than synthesis. Moreover, MUC15 overexpression resulted in accumulation of and high-molecular weight c-MET conjugates in liver T-ICs (Fig. 4G), suggesting that MUC15 promotes the degradation of c-MET through ubiquitination.

Notably, treatment with c-MET siRNA completely abolished the effects of MUC15 knockdown on p-AKT and SOX2 upregulation (Fig. 4H). Restoration of c-MET capitulated the expression of p-AKT and SOX2 in MUC15 overexpression HCC spheres (Fig. 4I). More importantly, the interference of c-MET abrogated the enhancement of self-renewal, colony formation, and tumorigenesis triggered by MUC15 knockdown in normal hepatocytes (Fig. 4J and Supplementary Fig. S5C, D). While introduction of c-MET recovered the self-renewal ability, liver T-ICs frequency and tumorigenesis capacity in MUC15 overexpression HCC spheres (Fig. 4K, L and Supplementary Fig. S5E).

### miR-183-5p.1 facilitates liver T-ICs expansion via targeting MUC15

**Fig. 5:**
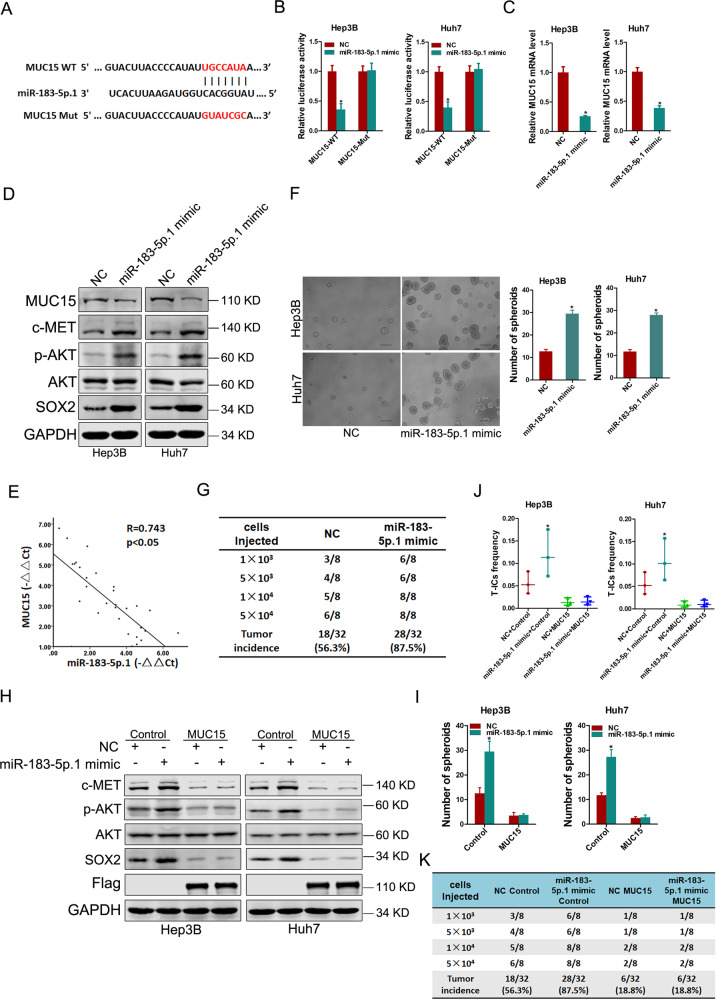
miR-183-5p.1 inhibits liver T-ICs expansion via targeting MUC15. **A** A potential target site for miR-183-5p.1 in the 3′UTR of human MUC15 mRNA, as predicted by the program TargetScan. To disrupt the interaction between miR-183-5p.1 and MUC15 mRNA, the target site was mutated. **B** Luciferase reporter assays performed in miR-183-5p.1 overexpression and control HCC spheres transfected with wild-type or mutant MUC15 3′UTR constructs. **C** Real-time PCR analysis of MUC15 in spheroids generated from miR-183-5p.1 overexpression hepatoma cells and control cells. **D** Western blot analysis of indicated proteins in spheroids generated from miR-183-5p.1 overexpression hepatoma cells and control cells. **E** The correlation between the level of MUC15 and miR-183-5p.1 in primary HCC cells (*n* = 30) was determined by real-time PCR analysis. **F** Representative images of hepatoma spheroids generated from miR-183-5p.1 overexpression hepatoma cells and control cells. The number of spheroids was counted and compared. **G** Hepatoma cells dissociated from Hep3B miR-183-5p.1 overexpression spheroids or control spheroids were inoculated into NOD-SCID mice subcutaneously, and the tumorigenicity was evaluated two months post inoculation. **H** Hep3B/Huh7 miR-183-5p.1 and control cells infected with MUC15 overexpression virus were subjected to western blot assays. **I** Hep3B/Huh7 miR-183-5p.1 and control cells infected with MUC15 overexpression virus were subjected to spheroids formation. **J** Hep3B/Huh7 miR-183-5p.1 and control cells infected with MUC15 overexpression virus were subjected to in vitro limiting dilution assays. **K** Hep3B miR-183-5p.1 and control cells infected with MUC15 overexpression virus were subjected to in vivo limiting dilution assays. Tumors were observed over 2 months; *n* = 8 for each group. All results are presented as the mean ± SD, and statistical significance was assessed using a two-tailed Student *t*-test. *p < 0.05.

To investigate the upstream regulatory mechanism of MUC15 in liver T-ICs, we searched the TargetScan database and identified candidate miRNA miR-183-5p.1. To confirm binding between miR-183-5p.1 and the MUC15 3′-UTR, we performed assays with a wild-type or mutated MUC15 3′-UTR-coupled luciferase reporter (Fig. 5A). Luciferase activity was suppressed by miR-183-5p.1 in HCC spheres transfected with wild-type MUC15 3′-UTR, mutation of which abrogated suppression (Fig. 5B). Real-time PCR and western blot further revealed that MUC15 was repressed in miR-183-5p.1-overexpressing HCC spheres (Fig. 5C, D). Consistent with this, levels of MUC15 and miR-183-5p.1 expression were negatively correlated in isolated primary HCC cells and HCC tumoral samples (Fig. 5E and Supplementary Fig. S6A).

Next, we found that the expression level of miR-183-5p was upregulated in HCC patients’ samples compared with the normal tissues (Supplementary Fig. S6B, C). Moreover, miR-183-5p.1 was upregulated in sorted EpCAM^+^ or CD24^+^ primary HCC cells and self-renewing spheroids (Supplementary Fig. S6D, E). In serial passages of primary HCC spheroids, miR-183-5p.1 expression gradually increased (Supplementary Fig. S6F). Notably, themiR-183-5p.1 level was recovered to the normal level when spheres became reattached (Supplementary Fig. S6G). Two HCC cell lines showed similar results (Supplementary Fig. S7H–K), further indicating that miR-183-5p.1 is generally upregulated in liver T-ICs. Furthermore, forced miR-183-5p.1 expression upregulated liver T-IC markers and pluripotent transcription factors, and enhanced spheroid formation (Fig. 5F and Supplementary Fig. S6L–N). In vitro and in vivo limiting dilution assays revealed that miR-183-5p.1 overexpression upregulated the proportion of liver T-ICs and their tumorigenesis capacity (Fig. 5G and Supplementary Fig. S6O).

We further investigated whether miR-183-5p.1 promotes liver T-ICs expansion by regulating MUC15 expression. We found that restoration of MUC15 abolished the effects of c-MET, p-AKT, and SOX2 upregulation in miR-183-5p.1 expressing cells (Fig. 5H). More importantly, introduction of MUC15 impaired the self-renewal ability, liver T-ICs frequency and tumorigenesis of miR-183-5p.1-overexpressing HCC spheres (Fig. 5I–K). Together, these data document that miR-183-5p.1 facilitates liver T-IC expansion by targeting MUC15.

### SOX2 upregulates miR-183-5p.1 in liver T-ICs

**Fig. 6:**
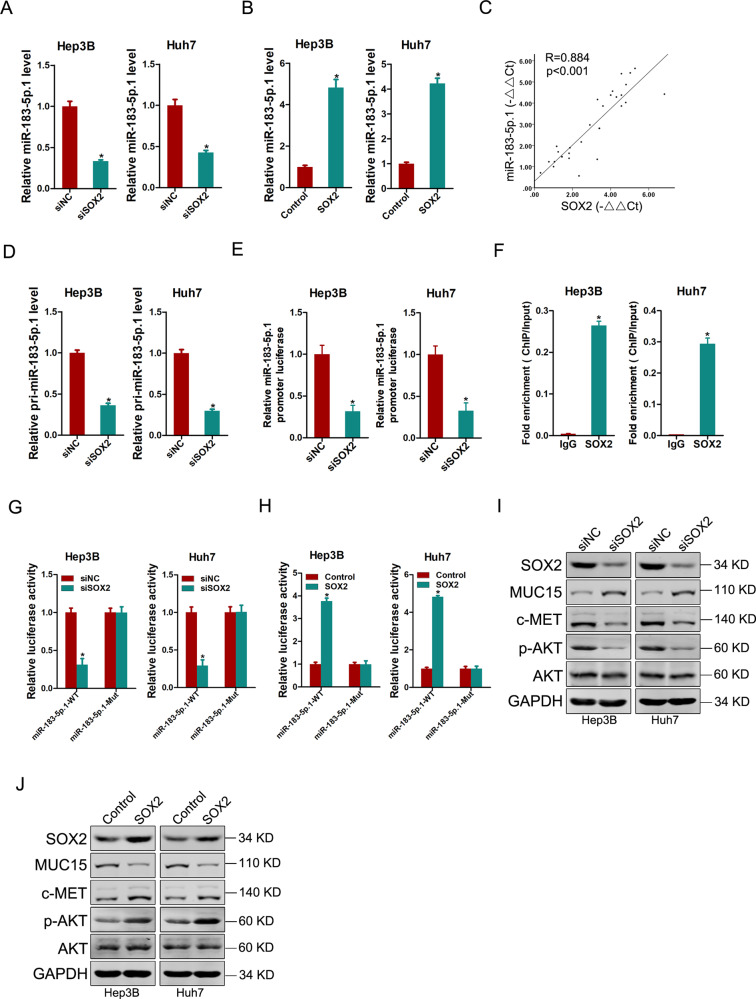
SOX2 upregulates miR-183-5p.1 in liver T-ICs. **A** Real-time PCR analysis of miR-183-5p.1 in spheroids generated from SOX2 knockdown hepatoma cells and control cells. **B** Real-time PCR analysis of miR-183-5p.1 in spheroids generated from SOX2 overexpression hepatoma cells and control cells. **C** The correlation between the level of SOX2 and miR-183-5p.1 in primary HCC cells (*n* = 30) was determined by real-time PCR analysis. **D** Real-time PCR analysis of pri-miR-183-5p.1 in spheroids generated from SOX2 knockdown hepatoma cells and control cells. **E** The luciferase reporter activity of miR-183-5p.1 promoter was measured in SOX2 knockdown and control HCC spheres. **F** Hepatoma cells subjected to ChIP assay with anti-SOX2 or anti-IgG antibody. **G** The luciferase reporter activity of miR-183-5p.1-WT or miR-183-5p.1-Mut promoter was measured in SOX2 knockdown and control HCC spheres, and the relative activity was presented (relative to control). **H** The luciferase reporter activity of miR-183-5p.1-WT or miR-183-5p.1-Mut promoter was measured in SOX2 overexpression and control HCC spheres, and the relative activity was presented (relative to control). **I** Western blot analysis of indicated proteins in spheroids generated from SOX2 overexpression hepatoma cells and control cells. **J** Western blot analysis of indicated proteins in spheroids generated from SOX2 knockdown hepatoma cells and control cells. All results are presented as the mean ± SD, and statistical significance was assessed using a two-tailed Student *t*-test. *p < 0.05.

Recently studies reported that SOX2 controls a network of coding and non-coding RNAs in cancers and identified a set of miRNAs as critical down-stream mediators of SOX2 function [21, 22]. In the present study, the levels of miR-183-5p.1 was decreased by SOX2 knockdown and increased upon SOX2 upregulation in HCC spheres (Fig. 6A, B). Consistently, a close correlation between SOX2 levels and miR-183-5p.1 expression was observed in isolated primary HCC cells, HCC tumoral samples and in liver T-ICs (Fig. 6C and Supplementary Fig. S7A, B). As shown in Fig. 6D and Supplementary Fig. S7C, forced SOX2 expression increased the level of pri-miR-183-5p.1 and SOX2 knockdown decreased the levels of pri-miR-183-5p.1 in HCC spheres. Furthermore, we found that the activation of the miR-183-5p.1 promoter was suppressed by knocking down SOX2, and enhanced by its ectopic expression (Fig. 6E and Supplementary Fig. S7D). ChIP assay further detected the significant enrichment of SOX2 in the promoter region of miR-183-5p.1 (Fig. 6F). Next, we found that mutation of the SOX2 binding site within the miR-183-5p.1 promoter region diminished the suppressive effect of SOX2 knockdown (Fig. 6G) or the enhancement effect on SOX2 overexpression (Fig. 6H). Furthermore, western blot analysis showed that MUC15 was downregulated and c-MET, p-AKT was upregulated in SOX2 knockdown hepatoma spheroids (Fig. 6I). Conversely, SOX2 overexpression decreased the level of MUC15 and increased the levels of c-MET, p-AKT in hepatoma spheroids (Fig. 6J), which further clarify that the MUC15/c-MET/PI3K/AKT/SOX2/miR-183-5p.1 regulatory circuit in liver T-ICs.

### MUC15 determines the chemotherapeutic response in HCC

**Fig. 7:**
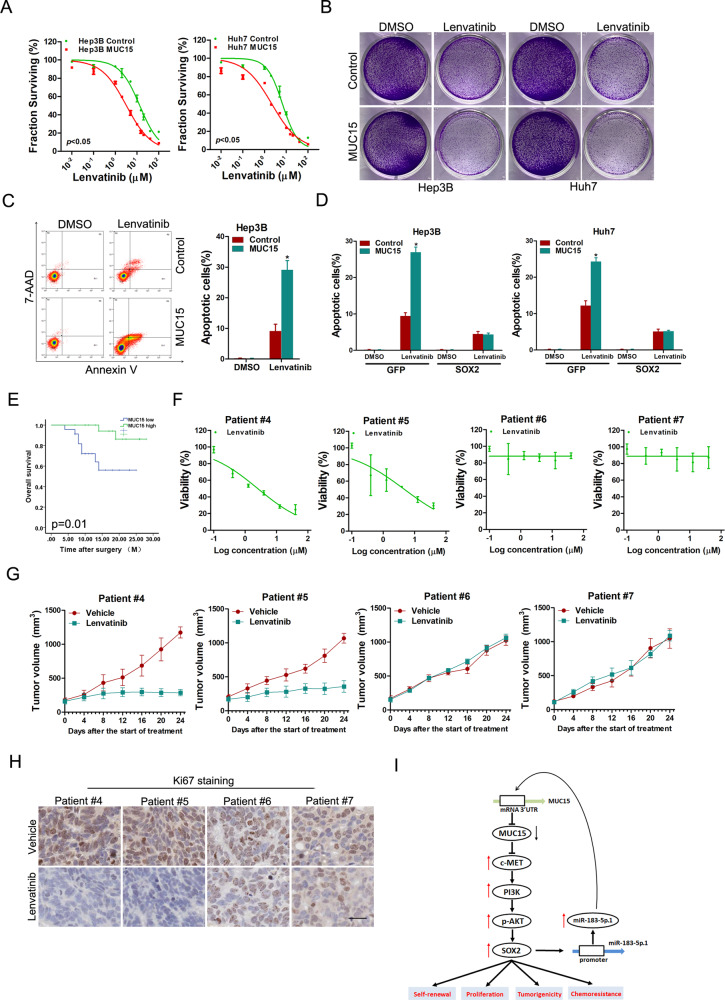
MUC15 determines the response of lenvatinib in HCC. **A** Hep3B/CSQT-2 MUC15 and control cells were treated with lenvatinib for 48 h and their cell survival curves was calculated. **B** Hep3B/CSQT-2 MUC15 and control cells were treated with lenvatinib (2.5 μM) for 7 days and their colony growth was examined. **C** Hep3B MUC15 and control cells were treated with lenvatinib (10 μM) for 48 h and their apoptosis was examined by flow cytometry. **D** MUC15 overexpression cells and control hepatoma cells infected with SOX2 overexpression virus were treated with lenvatinib for 48 h followed by cytometry analysis of apoptosis. **E** IHC staining and scoring of MUC15 expression was performed in HCC samples from 45 patients who had received adjuvant lenvatinib therapy after surgery. The overall survival (OS) of patients between MUC15-high or MUC15-low groups was evaluated by Kaplan–Meier analysis (*p* = 0.01). **F** PDOs derived from the primary HCCs with high MUC15 levels (Patient #4–5) or low MUC15 levels (Patient #6–7) were treated with lenvatinib for 7 days and their cell survival curves was calculated. **G** PDXs derived from the primary HCCs with high MUC15 levels (Patient #4–5) or low MUC15 levels (Patient #6–7) were treated with lenvatinib (60 mg/kg body weight) or vehicle for 24 days (*n* = 5 for each group). The xenograft growth was monitored. **H** PDXs derived from indicated patients treated with vehicle or lenvatinib were subjected to Ki67 staining. Representative views were shown. Scale bar = 25 μm. **I** A model showing miR-183-5p.1/MUC15/c-MET/PI3K/AKT/SOX2 regulatory circuit in manipulating the properties of liver tumor-initiating cells. All results are presented as the mean ± SD, and statistical significance was assessed using a two-tailed Student *t*-test. *p < 0.05.

Increasing evidence showed that liver T-ICs are closely associate with the resistance of cancer to pharmacotherapy [23]. As shown in Supplementary Fig. S8A, the expression of MUC15 was dramatically reduced in lenvatinib-resistant HCC PDXs and cell lines. The sensitivity of lenvatinib in hepatoma cells was upregulated in MUC15 overexpression hepatoma cells (Fig. 7A). We observed that MUC15 overexpression sensitized hepatoma cells to undergo lenvatinib-induced cell growth inhibition and cell apoptosis (Fig. 7B, C and Supplementary Fig. S8B, C). More importantly, introduction of SOX2, AKT or c-MET alleviated lenvatinib sensitivity in MUC15 overexpression hepatoma cells (Fig. 7D and Supplementary Fig. S8D, E).

We further investigate the clinical significance of MUC15 in patient response to lenvatinib therapy. HCC patients with high MUC15 expression displayed longer survival time following lenvatinib treatment than MUC15 low HCC patients (Fig. 7E). Next, by virtue of PDOs, we found that the PDOs derived from tumors with low MUC15 levels were resistant to lenvatinib treatment (Fig. 7F and Supplementary Fig. S8F, G). Similarly, the growth of PDXs derived from primary HCCs with high MUC15 levels was almost blocked upon lenvatinib treatment (Fig. 7G). Moreover, decreased expression of Ki67, a marker of proliferating cells, was only detected in lenvatinib-administrated PDXs derived from tumors with high MUC15 levels (Fig. 7H), suggesting that patient HCCs with high MUC15 expression could be more sensitive to lenvatinib treatment. These results further demonstrate that MUC15 levels in patient tumors might serve as a reliable predictor for lenvatinib response.

## Discussion

Despite advances in the research and treatment of HCC, the incidence and mortality of HCC have not improved. The existence of T-ICs prevents most HCC from being eradicated. However, the underlying regulatory mechanism for liver T-ICs is limited understanded. In this study, we found that miR-183-5p.1-mediated MUC15 mRNA degradation leads to downregulation of MUC15 expression in liver T-ICs, and MUC15 suppresses liver T-ICs properties and tumorigenicity via inhibition of c-MET/PI3K/AKT/SOX2 signaling. Moreover, we demonstrated that SOX2 feedback inhibits MUC15 expression by directly transactivating miR-183-5p.1, and forced of miR-183-5p.1 expression promoted liver T-ICs expansion via regulation of MUC15/c-MET/PI3K/AKT/SOX2 signaling (Fig. 7I). Our clinical investigations further demonstrated that MUC15 levels are associated with lenvatinib benefit in patients.

Although MUC15 was downregulated in HCC tissues and inhibited HCC metastasis, the role of MUC15 in liver T-ICs was not investigated. In this study, we identified that MUC15 inhibited liver oncogenesis via impairing liver T-ICs generation. In addition, we demonstrated that MUC15 levels was reduced in CD24^+^ or EpCAM^+^ cells as well as spheroid-enriched liver T-ICs. Functional experiments showed that MUC15 suppressed the self-renewal and expansion of liver T-ICs and thus inhibiting HCC initiation and progression. Together, our data indicate that MUC15 has an important function in liver T-ICs propagation, which also indicate that MUC15 is a potential therapeutic target.

The PI3K/AKT signaling pathway is involved in progression and activated in various cancers including HCC [24, 25]. Our RNA-seq results showed that PI3K/AKT signaling pathway inactivation was required for MUC15-mediated liver T-ICs expansion. Earlier studies have suggested that TIPMs and MMPs were participated in MUC15-mediated tumor metastasis [15]. However, we propose that MUC15 modulates liver T-ICs expansion via AKT signaling pathway inactivation and its downstream SOX2 restraint. Previous studies have elucidated that SOX2 plays vital roles in cancer cells proliferation, migration, and invasion [26]. In addition, SOX2 was reported to control tumor initiation and T-ICs functions [27]. In present study, we revealed that MUC15 downregulated SOX2 to inhibits T-ICs generation and weaken TIC-like properties of hepatoma cells, thus suppressing the initiation and progression of liver cancer. These discoveries not only provided a novel mechanism of liver T-ICs activation and also demonstrated the crucial role of MUC15/AKT/SOX2 axis in liver T-ICs.

c-MET has been reported to be associated with most human cancers, which can stimulate various downstream signaling pathways in tumor cells, such as PI3K/AKT, Wnt/β-catenin, JAK/STAT, Ras/MAPK, and SRC [28–30]. Our previous studies found that MUC15 was found to interact with EGFR to inhibit HCC metastasis [15]. In this study, our data suggest that MUC15 selectively modulates HGF-induced c-MET/PI3K/AKT signaling in liver T-ICs but has negligible impact on EGF-stimulated signaling. In addition, the physical interaction between MUC15 and c-MET effectively prevented the cell surface expression of c-MET and enhanced degradation of c-MET, which in turn modulated expression of PI3K/AKT/SOX2. Considering the important role of MUC15/c-MET/PI3K/AKT/SOX2 axis in liver T-ICs, we believe that targeting the MUC15/c-MET/ PI3K/AKT/SOX2 axis could be a novel therapeutic strategy for HCC.

Existing evidences are showing that miRNA expression is dysregulated in T-ICs and contribute to T-ICs expansion [8, 23]. We hypothesized that the loss or gain of miRNAs targeting MUC15 may cause downregulation of MUC15 in liver T-ICs. Our luciferase reporter assay identified miR-183-5p.1 as an upstream MUC15 regulator that directly targets MUC15. Dysregulated miR-183-5p.1 has been reported to function as an oncogene in gastric cancer [31]. In the present study, we demonstrated that miR-183-5p.1 was dramatically upregulated in liver T-ICs, and facilitates liver T-ICs self-renewal and tumorigenesis by targeting MUC15/c-MET/PI3K/AKT/SOX2 axis. Moreover, activated SOX2 upregulated the expression of miR-183-5p.1, thus completing an miR-183-5p.1/MUC15/c-MET/PI3K/AKT/SOX2 regulatory circuit. These findings not only provide a new mechanism of liver T-ICs activation and also implicate a crucial role of the miR-183-5p.1/MUC15/c-MET/PI3K/AKT/SOX2 axis in liver T-ICs.

Advanced HCC patients who develop resistance to lenvatinib have limited therapeutic options in the clinic at present [32–34]. Thus, it is urgent to investigate the biological basis of lenvatinib resistance and identify reliable biomarkers that can predict chemotherapeutic response in HCC patients. In the current study, we found that forced MUC15 expression sensitized HCC cells to lenvatinib-induced growth inhibition and apoptosis. More importantly, our data demonstrated that SOX2 is required for MUC15-mediated lenvatinib response in hepatoma cells. Therefore, targeting MUC15/c-MET/PI3K/AKT/SOX2 signaling could be an optimal combinational therapeutic strategy to overcome lenvatinib resistance in a subset of HCCs. Lenvatinib cohort, PDO and PDX studies further demonstrated that high MUC15 levels are associated with a better response and survival in patients, who have received lenvatinib treatment. Therefore, it is advisable to evaluate MUC15 expression in HCC tumors to identify patients who might benefit from lenvatinib therapy before deciding on a course of treatment, which merits further investigation in biomarker-guided clinical trials.

## Materials and methods

### Patients and analysis

All samples used in this study were obtained during liver transplantations or liver resections performed in the Eastern Hepatobiliary Surgery Hospital (EHBH) (Shanghai, China). Each High-grade dysplastic nodules (HGDN) specimen was diagnosed consistently by two senior pathologists and the criteria for HGDN reported previously [35]. Cohort 1 (*n* = 45) consists of HCC patients who received lenvatinib after hepatectomy in EHBH from 2018 to 2020. Detailed clinicopathological features are described in Supplementary Table S1. All of the above studies were approved by the Ethical Committee of the Second Military Medical University and conducted in accordance with the ethical guidelines of the World Medical Association Declaration of Helsinki.

### Cell lines and lentivirus

Normal liver cell lines HL7702 and THLE3, and HCC cell lines Hep3B and Huh7 were from Cell Bank of Type Culture Collection of Chinese Academy of Sciences (Shanghai Institute of Cell Biology of the Chinese Academy of Sciences). The HCC cells were cultured with Dulbecco’s modified Eagle’s medium (DMEM) supplemented with 10% and 15% fetal bovine serum (FBS) separately and 2 mM l-glutamine, and 25 µg/ml of gentamicin and maintained at 37 °C in 5% CO_2_ incubator. MUC15 knockdown, AKT overexpression, SOX2 overexpression, c-MET overexpression, miR-183-5p.1 overexpression, and control lentiviruses (designated as shMUC15, AKT, SOX2, c-MET, miR-183-5p.1 mimic, and GFP) were purchased from GenePharma (Shanghai, China). FLAG-MUC15 or control lentiviruses (designated as MUC15 and Control) were obtained from Obio Technology Co. (Shanghai, China). The anti-Flag antibody was used to recognize exogenous Flag-tagged MUC15 protein in Flag-tagged MUC15 infected cells.

### Mice and HCC induction

Muc15 knock-in (KI) mice and Muc15 flox/flox (KO) mice were generated by Beijing Biocytogen Co., Ltd (Beijing, China) by applying CRISPR/Cas9 system. Briefly, for Muc15 knock-in mice, a lox-stop-lox sequence followed by human Muc15 was inserted at Rosa26 locus (Rosa26-LSL-Muc15) using CRISPR/Cas9 system. The sequence of small guide RNA (sgRNA) was 5′-AAGGCCGCACCCTTCTCCGGAGG-3′. For Muc15 flox/flox mice, loxP sites flanking Muc15 exon 2 was inserted in C57BL/6 mouse using CRISPR/Cas9 system. The sequence of 5′ sgRNA was 5′-TCTCAATTAATCTGGACCCCTGG-3′ and the sequence of 3′ sgRNA was 5′-GTCACCCAATGTCTCAGGCAGGG-3′. Muc15 knock-in mice and Muc15 flox/flox mice were mated to Alb-Cre mice to generate liver specific Muc15 knock-in mice (ROSA26^Muc15^; Alb-Cre) and KO mice (Muc15^flox/flox^; Alb-Cre mice). All mice were given free access to food and water, and were maintained a 12/12 h light-dark cycle at 25 °C ambiences. For HCC induction, male mice were intraperitoneally injected with a single dose of diethylnitrosamine (DEN) (25 mg/kg body weight) at 14 days of age (*n* = 6 each, randomized allocated). At 4 weeks of age, mice then commenced intraperitoneally injection with CCl_4_ (0.5 ml/kg in olive oil, Shanghai Macklin Biochemical Co., Ltd) twice a week for up to 12 weeks and were sacrificed at the age of 5 months. The mouse livers and serum were collected for subsequent experiments. All animal procedures were conducted with the approval of the EHBH.

### In vivo xenograft and PDX model

BALB/c nude mice (male, 4 weeks old) were purchased from Chinese Academy of Sciences Slack Company (Shanghai, China). For in vivo tumor growth assay, Hep3B MUC15 and control spheroids were injected subcutaneously into six nude mice at 2 × 10^6^ cells per mouse (*n* = 8 each, randomized allocated). Xenografted tumor formation was monitored and the mice were sacrificed at 5 weeks of post inoculation. For the patient-derived xenograft (PDX) model, primary tumor samples were obtained for xenograft establishment as described previously [36]. When the PDX volume reaches approximately 100 mm^3^, nude mice were randomly assigned into lenvatinib group and len-control group. Mice in lenvatinib group were intraperitoneally injected with lenvatinib (60 mg/kg) or saline daily for 24 days (*n* = 5 for each group, randomized allocated). Tumor volumes were measured by caliper twice a week using formula Volume = *π*/6 * *L* * *W*^2^, where *L* is the longest tumor axis and *W* is the shortest tumor axis. When PDX volume reached approximately 1500 mm^3^, mice were euthanized by CO_2_, and tumor was sectioned or frozen for following analysis.

NOD-SCID mice (male, 4 weeks old) were purchased from Chinese Academy of Sciences Slack Company (Shanghai, China). For in vivo limiting dilution assay, spheroids were mixed with Matrigel (BD) at a ratio of 1:1 and injected subcutaneously at various cell doses per NOD-SCID mouse (*n* = 8 each, randomized allocated). Kinetic of tumor formation was evaluated per week for 8 weeks. For in vivo transformation assay, HL7702 cells infected with shMUC15 or GFP were dissociated into single cells and mixed with matrigel at a ratio of 1:1. The mixtures were then injected subcutaneously into eight NOD-SCID mice at 1 × 10^3^ cells per mouse (*n* = 6 each, randomized allocated). Xenografted tumor formation was monitored and the mice were sacrificed at 10 weeks of post-inoculation. All procedures and protocols were approved by the Ethics Committee of EHBH.

### Tissue dissociation and organoid culture

Organoid culture was performed as previously described [37]. Fresh liver cancer tissue was obtained, and the blood, fat, necrotic, and connective tissue on the tissue were cut off, and the area with abundant tumor cells was preserved and cut into pieces. The tissues were put into 5 ml 5 mM PBS/EDTA liquid for 15 min at room temperature. Then the tissues were placed in 5 mL of 1 mM PBS/EDTA containing 2× TrypLe and digested at 37 °C for 1 h. The cells are suspended in liquid by mechanical force blowing away the tissue mass. Isolated cells collected in DMEM/F12 medium, at 4 °C, 12,000 rpm centrifugal 5 min, make regrouped into granular cells. The granular cells suspended using 120 μl contains GFR matrigel and seeded into 24 or 48 cell culture plate. The drop was solidified by a 30 min incubation at 37 °C and 5% CO_2_. After solid drops formed, 1.5 ml of the organoid culture media was added to the well, and the medium was changed every 3–4 days. Lenvatinib with different concentrations were added to the organs when they grew to a certain amount and size.

### Data analysis

Statistical analysis was performed using SPSS V.18.0. The data are expressed as the mean ± SD. Student’s *t*-test or Mann–Whitney *U*-test was used to compare two variables and ANOVA to compare three or more. The patient survival of distinct subgroups was compared by Kaplan–Meier method and log-rank analysis. Pearson’s correlation analysis was performed to determine the correlation between two variables. Each data set was analyzed separately. A *p*-value less than 0.05 was considered statistically significant.

The remainder of the description of the materials and methods can be found in the Supplementary materials.

## Supplementary information

MUC15 supplementary

original western blots

aj-checklist

## Data Availability

The data in the current study are available from the corresponding authors upon reasonable request.

## References

[1] Siegel RL, Miller KD, Fuchs HE, Jemal A (2021). Cancer statistics, 2021. Cancer J Clin.

[2] Xiang D, Cheng Z, Liu H, Wang X, Han T, Sun W (2017). Shp2 promotes liver cancer stem cell expansion by augmenting beta-catenin signaling and predicts chemotherapeutic response of patients. Hepatology.

[3] Zhou T, Li S, Xiang D, Liu J, Sun W, Cui X (2020). m6A RNA methylation-mediated HNF3gamma reduction renders hepatocellular carcinoma dedifferentiation and sorafenib resistance. Signal Transduct Target Ther.

[4] Lapidot T, Sirard C, Vormoor J, Murdoch B, Hoang T, Caceres-Cortes J (1994). A cell initiating human acute myeloid leukaemia after transplantation into SCID mice. Nature.

[5] Clevers H (2011). The cancer stem cell: premises, promises and challenges. Nat Med.

[6] O’Brien CA, Pollett A, Gallinger S, Dick JE (2007). A human colon cancer cell capable of initiating tumour growth in immunodeficient mice. Nature.

[7] Collins AT, Berry PA, Hyde C, Stower MJ, Maitland NJ (2005). Prospective identification of tumorigenic prostate cancer stem cells. Cancer Res.

[8] Li L, Tang J, Zhang B, Yang W, LiuGao M, Wang R (2015). Epigenetic modification of MiR-429 promotes liver tumour-initiating cell properties by targeting Rb binding protein 4. Gut.

[9] Pallesen LT, Berglund L, Rasmussen LK, Petersen TE, Rasmussen JT (2002). Isolation and characterization of MUC15, a novel cell membrane-associated mucin. Eur J Biochem.

[10] Shyu MK, Lin MC, Shih JC, Lee CN, Huang J, Liao CH (2007). Mucin 15 is expressed in human placenta and suppresses invasion of trophoblast-like cells in vitro. Hum Reprod.

[11] Wang S, Li J, You L, Dai M, Zhao Y (2020). High expression of MUC15 is correlated with poor prognosis of pancreatic cancer and promotes migration, invasion, and chemo-resistance in vitro. Med Sci Monit.

[12] Dai W, Liu J, Liu B, Li Q, Sang Q, Li YY (2020). Systematical analysis of the Cancer Genome Atlas Database reveals EMCN/MUC15 combination as a prognostic signature for gastric cancer. Front Mol Biosci.

[13] Huang J, Che MI, Huang YT, Shyu MK, Huang YM, Wu YM (2009). Overexpression of MUC15 activates extracellular signal-regulated kinase 1/2 and promotes the oncogenic potential of human colon cancer cells. Carcinogenesis.

[14] Choi C, Thi Thao Tran N, Van Ngu T, Park SW, Song MS, Kim SH (2018). Promotion of tumor progression and cancer stemness by MUC15 in thyroid cancer via the GPCR/ERK and integrin-FAK signaling pathways. Oncogenesis.

[15] Wang RY, Chen L, Chen HY, Hu L, Li L, Sun HY (2013). MUC15 inhibits dimerization of EGFR and PI3K-AKT signaling and is associated with aggressive hepatocellular carcinomas in patients. Gastroenterology.

[16] Yang Y, Lin X, Lu X, Luo G, Zeng T, Tang J (2016). Interferon-microRNA signalling drives liver precancerous lesion formation and hepatocarcinogenesis. Gut.

[17] Lee TK, Castilho A, Cheung VC, Tang KH, Ma S, Ng IO (2011). CD24(+) liver tumor-initiating cells drive self-renewal and tumor initiation through STAT3-mediated NANOG regulation. Cell Stem Cell.

[18] Yamashita T, Ji J, Budhu A, Forgues M, Yang W, Wang HY (2009). EpCAM-positive hepatocellular carcinoma cells are tumor-initiating cells with stem/progenitor cell features. Gastroenterology.

[19] Wang Z, Kang L, Zhang H, Huang Y, Fang L, Li M (2019). AKT drives SOX2 overexpression and cancer cell stemness in esophageal cancer by protecting SOX2 from UBR5-mediated degradation. Oncogene.

[20] Quan MY, Guo Q, Liu J, Yang R, Bai J, Wang W (2020). An FGFR/AKT/SOX2 signaling axis controls pancreatic cancer stemness. Front Cell Dev Biol.

[21] Liu K, Xie F, Gao A, Zhang R, Zhang L, Xiao Z (2017). SOX2 regulates multiple malignant processes of breast cancer development through the SOX2/miR-181a-5p, miR-30e-5p/TUSC3 axis. Mol Cancer.

[22] Yang Y, Fan X, Ren Y, Wu K, Tian X, Wen F (2021). SOX2-upregulated microRNA-30e promotes the progression of esophageal cancer via regulation of the USP4/SMAD4/CK2 axis. Mol Ther Nucleic Acids.

[23] Han T, Zhang Y, Yang X, Han L, Li H, Chen T (2020). miR-552 regulates liver tumor-initiating cell expansion and sorafenib resistance. Mol Ther Nucleic Acids.

[24] Chen J, Ge X, Zhang W, Ding P, Du Y, Wang Q (2020). PI3K/AKT inhibition reverses R-CHOP resistance by destabilizing SOX2 in diffuse large B cell lymphoma. Theranostics.

[25] Han T, Xiang DM, Sun W, Liu N, Sun HL, Wen W (2015). PTPN11/Shp2 overexpression enhances liver cancer progression and predicts poor prognosis of patients. J Hepatol.

[26] Bass AJ, Watanabe H, Mermel CH, Yu S, Perner S, Verhaak RG (2009). SOX2 is an amplified lineage-survival oncogene in lung and esophageal squamous cell carcinomas. Nat Genet.

[27] Schaefer T, Lengerke C (2020). SOX2 protein biochemistry in stemness, reprogramming, and cancer: the PI3K/AKT/SOX2 axis and beyond. Oncogene.

[28] Zhang Y, Xia M, Jin K, Wang S, Wei H, Fan C (2018). Function of the c-Met receptor tyrosine kinase in carcinogenesis and associated therapeutic opportunities. Mol Cancer.

[29] Papa E, Weller M, Weiss T, Ventura E, Burghardt I, Szabo E (2017). Negative control of the HGF/c-MET pathway by TGF-beta: a new look at the regulation of stemness in glioblastoma. Cell Death Dis.

[30] El Bezawy R, De Cesare M, Pennati M, Deraco M, Gandellini P, Zuco V (2017). Antitumor activity of miR-34a in peritoneal mesothelioma relies on c-MET and AXL inhibition: persistent activation of ERK and AKT signaling as a possible cytoprotective mechanism. J Hematol Oncol.

[31] Lin J, Shen J, Yue H, Cao Z (2019). miRNA1835p.1 promotes the migration and invasion of gastric cancer AGS cells by targeting TPM1. Oncol Rep.

[32] Kudo M, Finn RS, Qin S, Han KH, Ikeda K, Piscaglia F (2018). Lenvatinib versus sorafenib in first-line treatment of patients with unresectable hepatocellular carcinoma: a randomised phase 3 non-inferiority trial. Lancet.

[33] Tohyama O, Matsui J, Kodama K, Hata-Sugi N, Kimura T, Okamoto K (2014). Antitumor activity of lenvatinib (e7080): an angiogenesis inhibitor that targets multiple receptor tyrosine kinases in preclinical human thyroid cancer models. J Thyroid Res.

[34] Ikeda K, Kudo M, Kawazoe S, Osaki Y, Ikeda M, Okusaka T (2017). Phase 2 study of lenvatinib in patients with advanced hepatocellular carcinoma. J Gastroenterol.

[35] Roncalli M, Borzio M, Di Tommaso L (2007). Hepatocellular dysplastic nodules. Hepatol Res.

[36] Xiang DM, Sun W, Zhou T, Zhang C, Cheng Z, Li SC (2019). Oncofetal HLF transactivates c-Jun to promote hepatocellular carcinoma development and sorafenib resistance. Gut.

[37] Lee SH, Hu W, Matulay JT, Silva MV, Owczarek TB, Kim K (2018). Tumor evolution and drug response in patient-derived organoid models of bladder. Cancer Cell.

