# Supplementary material for: Downregulation of MUC15 by miR-183-5p.1 promotes liver tumor-initiating cells properties and tumorigenesis via regulating c-MET/PI3K/AKT/SOX2 axis
Source: Cell Death Dis. 2022 Mar 2;13(3):200. doi: 10.1038/s41419-022-04652-9 (PMC8891362; doi:10.1038/s41419-022-04652-9)
Supplement: Supplementary file 1 — MUC15 supplementary [file 41419_2022_4652_MOESM1_ESM.docx]

**Downregulation of MUC15 by miR-183-5p.1 promotes liver tumor-initiating cells properties and tumorigenesis via regulating c-MET/PI3K/AKT/SOX2 axis**

Tao Han^1,#^, Hao Zheng^2,3,4,5,#^, Jin Zhang^2,#^, Pinghua Yang^2,#^, Hengyu Li^6,*^, Zhangjun Cheng^7,*^, Daimin Xiang^8, *^, Ruoyu Wang^2,*^

^1^Department of Oncology, the First Affiliated Hospital of China Medical University, Shenyang 110001, China;

^2^Department of Hepatic Surgery, Third Affiliated Hospital of Second Military Medical University, Shanghai, 200438, China;

^3^Key Laboratory of Signaling Regulation and Targeting Therapy of Hepatocellular Carcinoma Ministry of Education, Shanghai, 200438, China;

^4^Shanghai Key Laboratory of Hepatobiliary Tumor Biology, Shanghai, 200438, China; ^5^Department of Reproductive Heredity Center, Changhai Hospital, Second Military Medical University, Shanghai, 200433, People's Republic of China.

^6^Department of General Surgery, Changhai Hospital, Second Military Medical University, Shanghai, 200438, China;

^7^Department of Hepato-pancreato-biliary centers, Zhong Da Hospital, School of Medicine, Southeast University, Nanjing, 210009, China;

^8^State Key Laboratory of Oncogenes and Related Genes, Shanghai Cancer Institute, Renji Hospital, Shanghai Jiao Tong University School of Medicine, Shanghai, 200127, China;

^#^The authors have contributed equally to this work.

***Correspondence author address:**

Dr. Ruoyu Wang: Department of Hepatic Surgery, Third Affiliated Hospital of Second Military Medical University, Shanghai, 200438, China; Email: ruoyuwang202202@163.com; Dr. Daimin Xiang: State Key Laboratory of Oncogenes and Related Genes, Shanghai Cancer Institute, Renji Hospital, Shanghai Jiao Tong University School of Medicine, Shanghai, 200127, China; Email: xdm20079@126.com; Dr. Zhangjun Cheng: Department of Hepato-pancreato-biliary centers, Zhong Da Hospital, School of Medicine, Southeast University, Nanjing, 210009, China; Email: [chengzhangjun@seu.edu.cn](mailto:chengzhangjun@seu.edu.cn). Dr. Hengyu Li: Department of General Surgery, Zhong Da Hospital, First Affiliated Hospital of Second Military Medical University, Shanghai, 200433, China; Email: [lhy@smmu.edu.cn](mailto:lhy@smmu.edu.cn).

**Materials and Methods**

***Spheroids assay***

Three hundred single cells were seeded into 96-well Ultra-Low Attachment Microplates (Corning, USA) in serum-free DMEM/F12 (Invitrogen, USA), supplemented with B27 (1:50, Invitrogen), 20 ng/ml EGF (Peprotech), 10 ng/ml bFGF (Invitrogen), and 4 mg/ml insulin (Sigma). Spheres were photographed and counted 7 days after seeding (primary spheres).

***In vitro limiting dilution assay***

Hepatoma cells were seeded into 96-well ultra-low attachment culture plates at various cell numbers and incubated for 7 days. Based on the frequency of wells with spheres forming, the proportion of tumor-initiating cells was determined using Poisson distribution statistics and the LCalc Version 1.1 software program (Stem Cell Technologies, Inc. Vancouver, Canada) [1].

***Immunohistochemistry and immunofluorescent staining***

The tissue samples were fixed with 10% neutral formaldehyde and embedded in paraffin, and sectioned for hematoxylin-eosin (H&E) staining or immunohistochemical (IHC) staining as described previously [2]. In brief, after antigen retrieval, sections or tissue microarray (TMA) were blocked by bovine serum antigen albumin and incubated with indicated primary antibody and then secondary antibody. A diaminobenzidine colorimetric reagent solution was used, followed by hematoxylin counterstaining. The slides were scanned and representative images were captured. IHC scoring was based on the percentage of positively stained cells and staining intensity assessed by Image-scope software (Aperio Technologies, Inc.). Immunofluorescent staining was performed using a tyramide signal amplification (TSA) fluorescence kit (TSA Plus Fluorescein, NEL741001KT; PerkinElmer, Waltham, Massachusetts, USA) according to the manufacturer’s instructions. The antibodies used for IHC or immunofluorescent staining are listed in online Supplementary table 3.

***Flow cytometric analysis***

To analyze the proportion of EpCAM or CD24-positive liver CSCs, the hepatoma cells were harvested and resuspended in the staining buffer, then incubated with APC-conjugated EpCAM antibody (BioLegend) or CD24 antibody (BioLegend) for 30 minutes at 4℃ in the dark. The cells were further washed with cold staining buffer twice and resuspended in the staining buffer containing 1 μg/mL propidium iodide (PI, BioLegend) followed by flow cytometry analysis using a Moflo XDP flow cytometer from Beckman Coulter.

For CD24^+^ or EPCAM^+^ cell sorting, hepatoma cells (5×10^7^) were harvested and resuspended in cold staining buffer, then incubated with antibody against human CD24 (BioLegend) or EpCAM (BioLegend), respectively. Positively and negatively stained cells were then sorted by Moflo XDP flow cytometer. The sorted cells from three independent experiments were subjected to Real-time PCR assay.

***RNA interference***

Small interference RNAs (siRNAs) against c-MET or SOX2 and NC (NC, negative control) siRNA were synthetized by Genepharma (Shanghai, China). siRNA target sequences are listed in online Supplementary table 4. The siRNAs were transfected into the hepatoma cells at a final concentration of 200 nM using lipofectamine 2000 Transfection Reagen according to the manufacturer’s instructions (Thermo Fisher Scientific). The cells were harvested or subjected to further downstream experiments 24-72 hours after transfection.

***RNA sequencing***

Total RNA (Hep3B MUC15 and control spheroids) was extracted using the mirVana miRNA Isolation Kit (Ambion) following the manufacturer’s protocol. RNA integrity was evaluated using the Agilent 2100 Bioanalyzer (Agilent Technologies, Santa Clara, CA, USA). The samples with RNA Integrity Number (RIN) ≥ 7 were subjected to the subsequent analysis. The libraries were constructed using TruSeq Stranded mRNA LTSample Prep Kit (Illumina, San Diego, CA, USA) according to the manufacturer’s instructions. Then these libraries were sequenced on the Illumina sequencing platform (HiSeqTM 2500 or Illumina HiSeq X Ten) and 125bp/150bp paired-end reads were generated.

The transcriptome sequencing and analysis were conducted by OE biotech CO., Ltd. (Shanghai China). Raw data (ram reads) were processed using Trimmomatic. The reads containing poly-N and the low-quality reads were removed to obtain the clean reads. Then the clean reads were mapped to reference genome using hisat2. FPKM value of each gene was calculated using cufflinks, and the read counts of each gene were obtained by htseq-count. DEGs were identified using the DE Seq (2012) R package functions estimate Size Factors and nbinom Test. P value < 0.05 and fold Change > 2 or fold Change < 0.5 was set as the threshold for significantly differential expression. Hierarchical cluster analysis of DEGs was performed to explore genes expression pattern. Gene ontology and gene set enrichment analysis of DEGs were respectively performed using R based on the hypergeometric distribution.

***Western blot analysis***

Western blot analysis was performed as previously described [3]. Protein extracts of HCC cells or mouse HCC tissues were analyzed by immunoblot with the primary antibody and an IRDye 800CW-conjugated secondary antibody, and then scanned by LI-COR imaging system (LI-COR Biosciences). The antibodies used for Western blot are listed in supplementary table S2.

***Real-time PCR analysis***

Total RNA was extracted from tissues or cells using TRIzol (Invitrogen) and was reverse transcribed using a Reverse Transcription System (Promega) to synthesize cDNA. In Roche Light Cycler 96 System (Roche, USA), the cDNA was mixed with SYBR Green PCR Kit (Roche) and specific primers to actuate real time PCR. PCR conditions included 1 cycle at 95 °C for 5 minutes, followed by up to 40 cycles of 95 °C for 15 seconds (denaturation), 60 °C for 30 seconds (annealing) and 72 °C for 30 seconds (extension). The sequences of primers used were listed in supplementary table S3. The specificity of primers was confirmed by melting curves following the reaction. Each sample was measured in triplicate biological replicates. Each experiment was repeated at least three times and the representative results were shown.

***Immunoprecipitation (IP) analysis***

Hepatoma cells were harvested by IP lysis buffer supplemented with a protease inhibitor cocktail (Sigma-Aldrich, St. Louis, MO). The cell lysates were centrifuged at 4 °C at 12000g for 15 min and the supernatant was quantified. The supernatant was incubated with appropriate primary antibodies overnight, followed by incubating with protein A/G PLUS-Agarose beads (Santa Cruz Biotech., sc-2003) for 4 hours. After washing for three times using ice-cold lysis buffer, beads were boiled with 5× loading buffer for 5 min followed by SDS-PAGE and western blotting using the appropriate primary and second antibodies.

***Colony formation assays***

Colony formation assay was used to assess the transformation activity of cells. Briefly, 1 mL of 0.66% soft agar solution was poured into a 12-well plate and solidified at room temperature. For the top layer, 1 mL of 0.33% agar solution containing 3×10^3^ cells with 10% FBS was poured. An extra 1 mL of cell culture medium was added. The cells were cultured for three weeks and the colonies formed were counted through microscope.

***PI3K activity assay***

PI3K from HCC spheroid cells was immunoprecipitated with an anti-p85α antibody and protein A/G PLUS-agarose beads (Santa Cruz Biotechnology, Dallas, TX, USA). PI3K activity in the immunoprecipitates was analyzed with a PI3K enzyme-linked immunosorbent assay (ELISA) kit (Echelon Biosciences, Salt Lake City, UT, USA) according to the manufacturer’s instructions.

**Reference:**

1. Qu L, Wu Z, Li Y, Xu Z, Liu B, Liu F, et al., *A feed-forward loop between lncARSR and YAP activity promotes expansion of renal tumour-initiating cells.* Nat Commun, 2016. **7**: p. 12692.

2. Wang X, Sun W, Shen W, Xia M, Chen C, Xiang D, et al., *Long non-coding RNA DILC regulates liver cancer stem cells via IL-6/STAT3 axis.* J Hepatol, 2016. **64**(6): p. 1283-94.

3. Xiang DM, Sun W, Ning BF, Zhou TF, Li XF, Zhong W, et al., *The HLF/IL-6/STAT3 feedforward circuit drives hepatic stellate cell activation to promote liver fibrosis.* Gut, 2018 09;67(9).


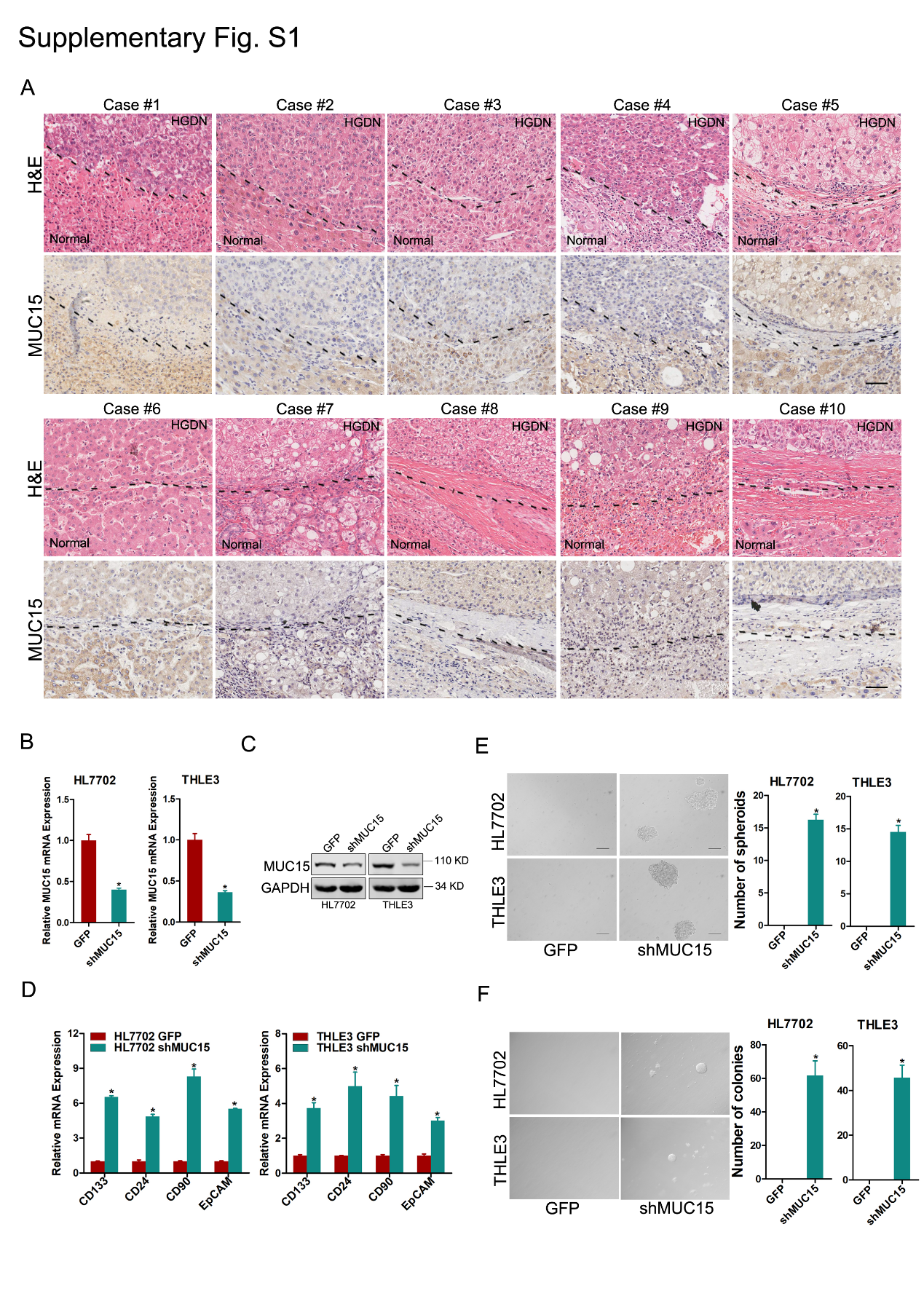


**Supplementary Fig. S1**

**A.** H&E staining and IHC for MUC15 in human HGDN and paired adjacent normal liver tissues. Scale bar=50 μm.

**B.** HL7702 and THLE3 cells infected with MUC15 knockdown virus were subjected to real-time PCR analysis.

**C.** HL7702 and THLE3 cells infected with MUC15 knockdown virus were subjected to western blot analysis.

**D.** shMUC15 and control hepatocytes were subjected to real-time PCR analysis.

**E.** shMUC15 and control hepatocytes were subjected to spheroid formation assays.

**F.** shMUC15 and control hepatocytes were subjected to colony formation assays.


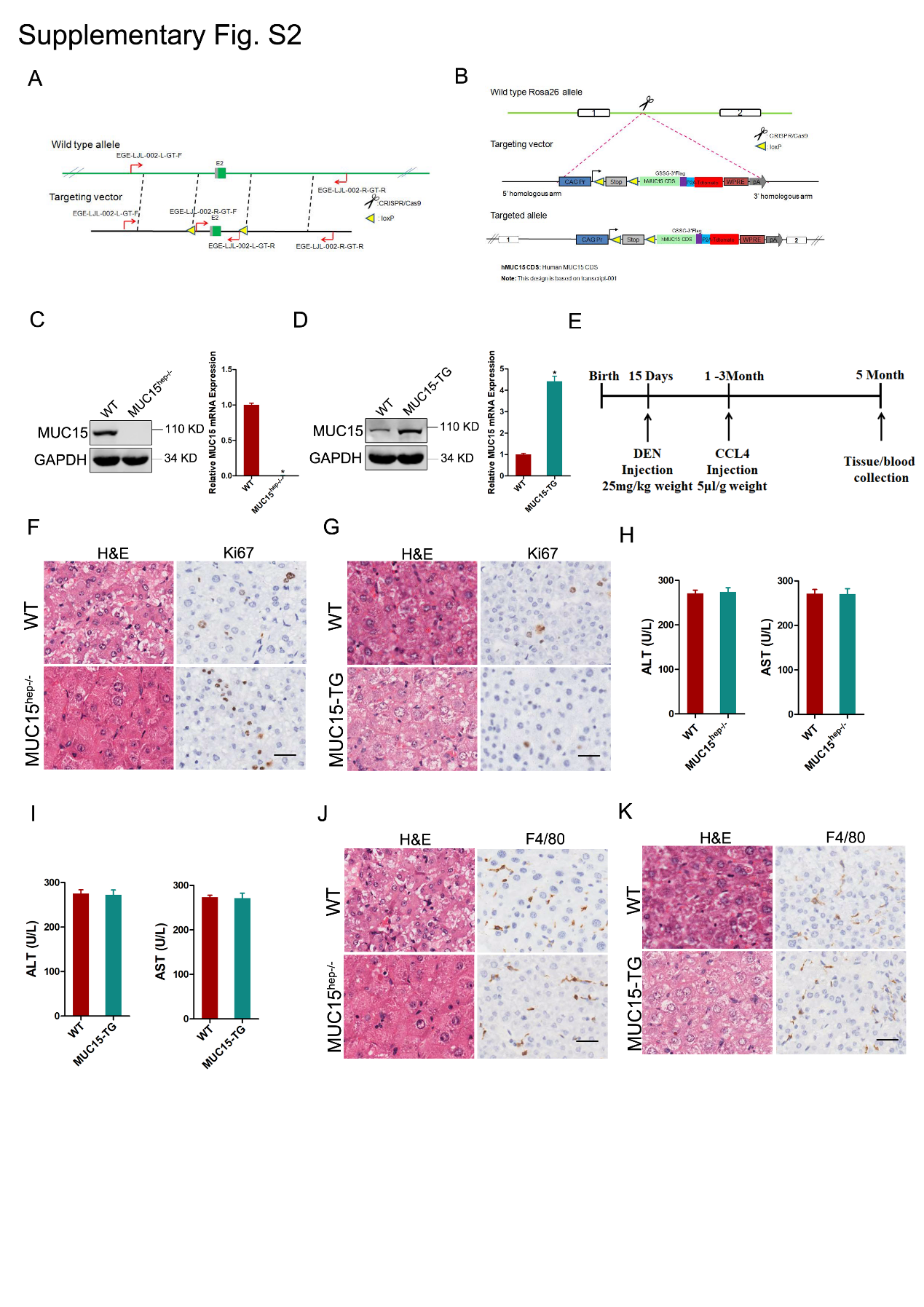


**Supplementary Fig. S2**

**A&B.** Schematics of MUC15^hep-/-^ and MUC15-TG mice mice.

**C.** Real-time PCR and western blot analysis of MUC15 levels in livers from MUC15^hep-/-^ and WT mice.

**D.** Real-time PCR and western blot analysis of MUC15 levels in livers from MUC15-TG and WT mice.

**E.** HCC mouse model design.

**F.** Representative images of H&E and IHC staining of Ki67 in liver tumors of MUC15^hep-/-^ and WT mice.

**G.** Representative images of H&E and IHC staining of Ki67 in liver tumors of MUC15-TG and WT mice.

**H.** The serum ALT and AST levels of MUC15^hep-/-^ and WT mice at 5 months after DEN injection were examined.

**I.** The serum ALT and AST levels of MUC15-TG and WT mice at 5 months after DEN injection were examined.

**J.** Representative images of H&E and IHC staining of F4/80 in liver tumors of MUC15^hep-/-^ and WT mice.

**K.** Representative images of H&E and IHC staining of F4/80 in liver tumors of MUC15-TG and WT mice.


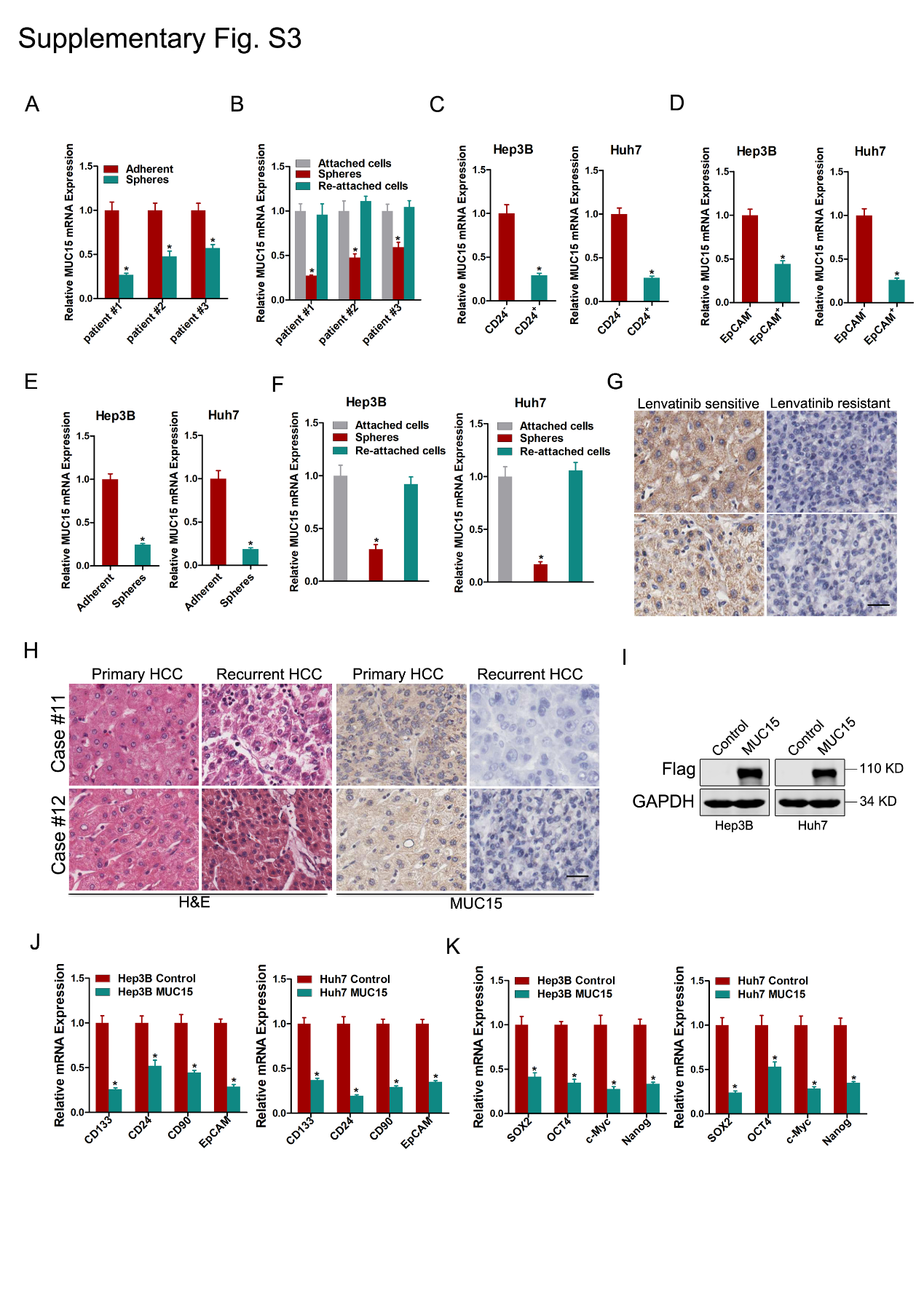


**Supplementary Fig. S3**

**A.** Real-time PCR analysis of MUC15 expression in primary HCC adherent cells and spheres.

**B.** Real-time PCR analysis of MUC15 expression in primary HCC adherent, spheres and re-adherent cells.

**C.** Real-time PCR analysis MUC15 expression in sorted CD24^+^ HCC cells relative to negative cells.

**D.** Real-time PCR analysis MUC15 expression in sorted EpCAM^+^ HCC cells relative to negative cells.

**E.** Real-time PCR analysis of MUC15 expression in HCC adherent cells and spheres.

**F.** Real-time PCR analysis of MUC15 expression in HCC adherent, spheres and re-adherent cells.

**G.** Representative images of IHC staining of MUC15 in lenvatinib-sensitive or lenvatinib-resistant HCC tissues. Scale bar=25 μm.

**H.** Representative images of H&E and IHC staining of MUC15 in recurrent HCC and primary lesions of patient. Scale bar=25 μm.

**I.** HCCLM3 and Huh7 cells infected with MUC15 overexpression virus were subjected to western blot analysis.

**J&K.** Real-time PCR analysis of liver T-IC markers or stemness-associated transcription factors in spheroids generated from MUC15 overexpression hepatoma cells and control cells.


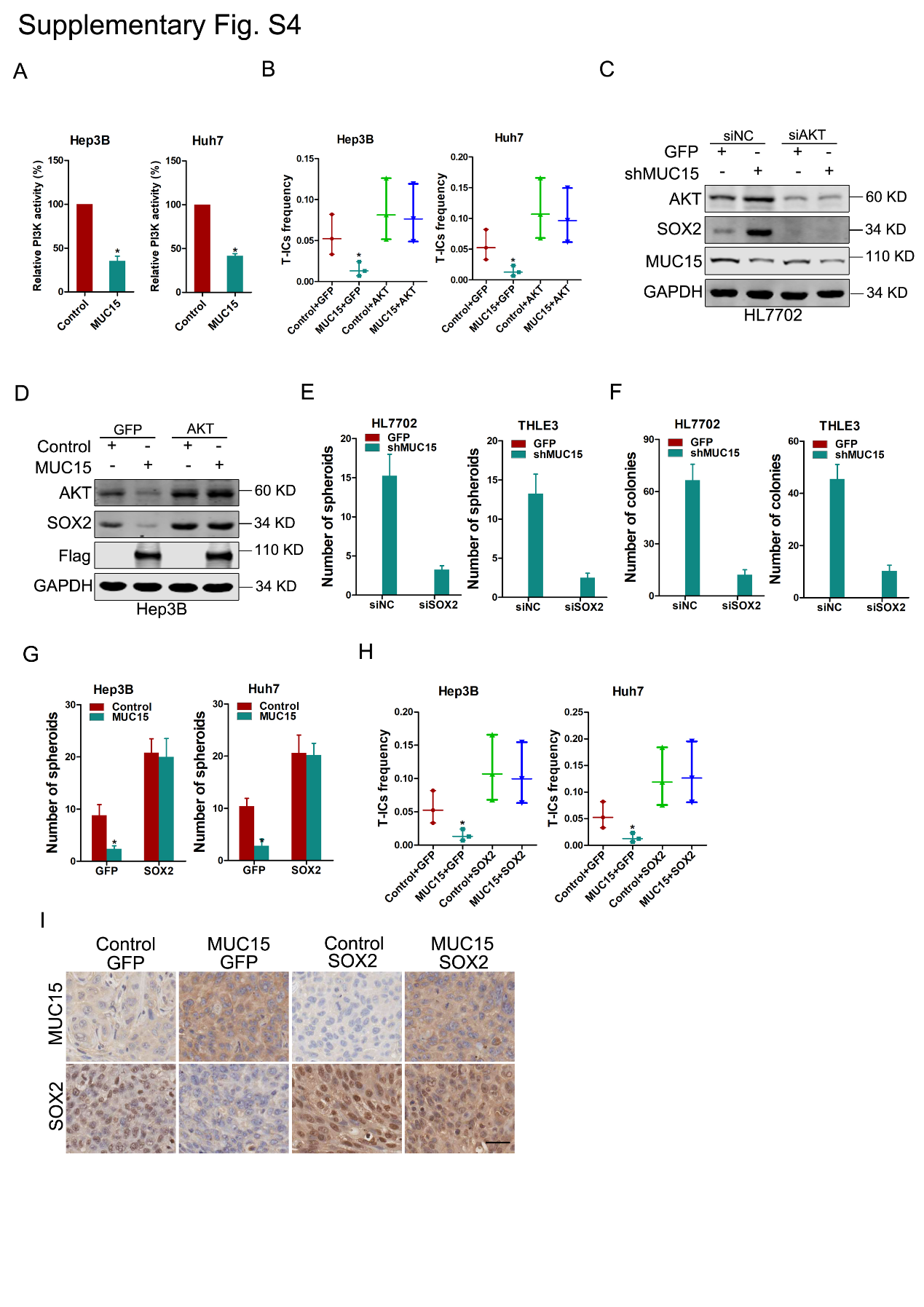


**Supplementary Fig. S4**

**A.** The PI3K kinase activity was determined as described in the Methods section.

**B.** Hep3B MUC15 and control cells infected with AKT overexpression virus were subjected to *in vitro* limiting dilution assay.

**C.** HL7702 shMUC15 and control cells transfected with siSOX2 or siNC were subjected to western blot analysis.

**D.** Hep3B MUC15 and control cells infected with AKT overexpression virus were subjected to western blot analysis.

**E.** HL7702/THLE3 shMUC15 and control cells were transfected with siSOX2 or siNC and were then subjected to spheroids formation assay.

**F.** HL7702/THLE3 shMUC15 and control cells were transfected with siSOX2 or siNC and were then colony formation assay.

**G.** Hep3B/CSQT-2 MUC15 and control cells were infected with SOX2 overexpression virus and were then subjected to spheroids formation assay.

**H.** Hep3B/CSQT-2 MUC15 and control cells were infected with SOX2 overexpression virus and were then subjected to *in vitro* limiting dilution assay.

**I.** Immunohistochemical staining of MUC15 and SOX2 in xenografted tumors generated by indicated HCC cells. Scale bar=25 μm.


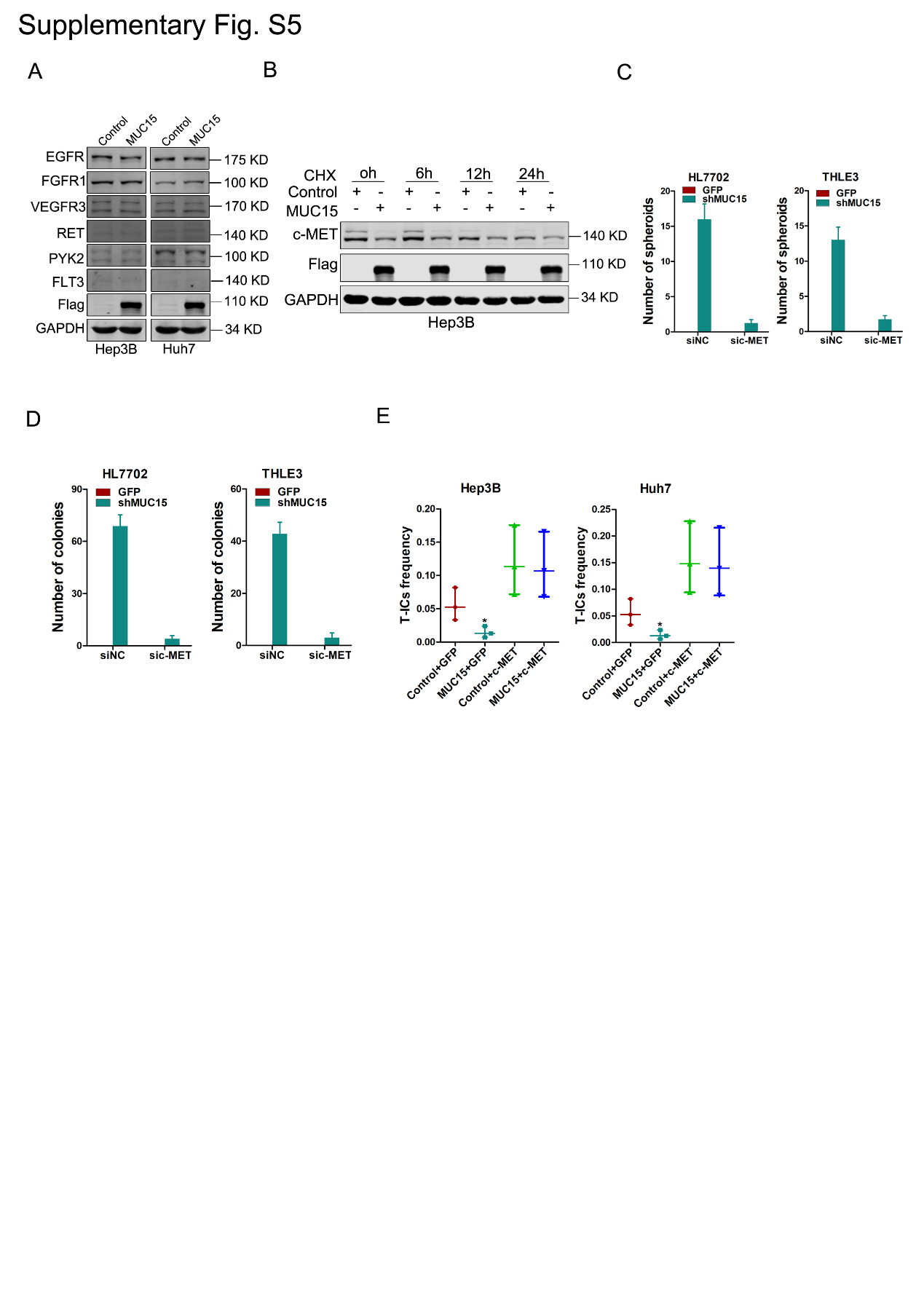


**Supplementary Fig. S5**

**A.** The expression of EGFR, FGFR1, VEGFR3, RET, PYK2 or FLT3 in HCCLM3/CSQT-2 MUC15 and control spheroids was determined by western blot.

**B.** Hep3B MUC15 and control spheroids were treated with CHX (100 μg/ml) for indicated times and then subjected to western-blot analysis.

**C.** HL7702/THLE3 shMUC15 and control cells were transfected with sic-MET or siNC and were then subjected to spheroids formation assay.

**D.** HL7702/THLE3 shMUC15 and control cells were transfected with sic-MET or siNC and were then colony formation assay.

**E.** Hep3B/CSQT-2 MUC15 and control cells were infected with c-MET overexpression virus and were then subjected to *in vitro* limiting dilution assay.


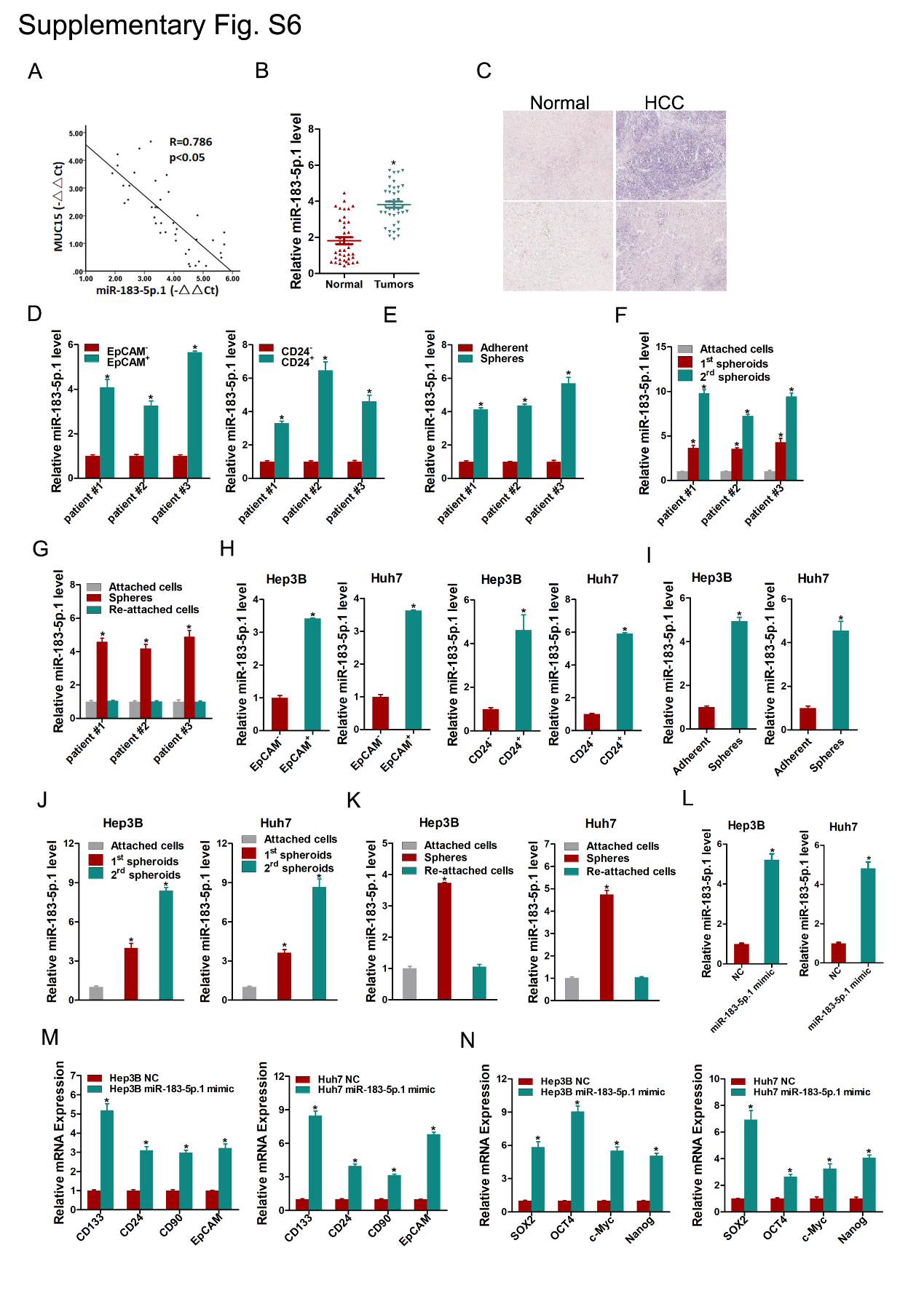

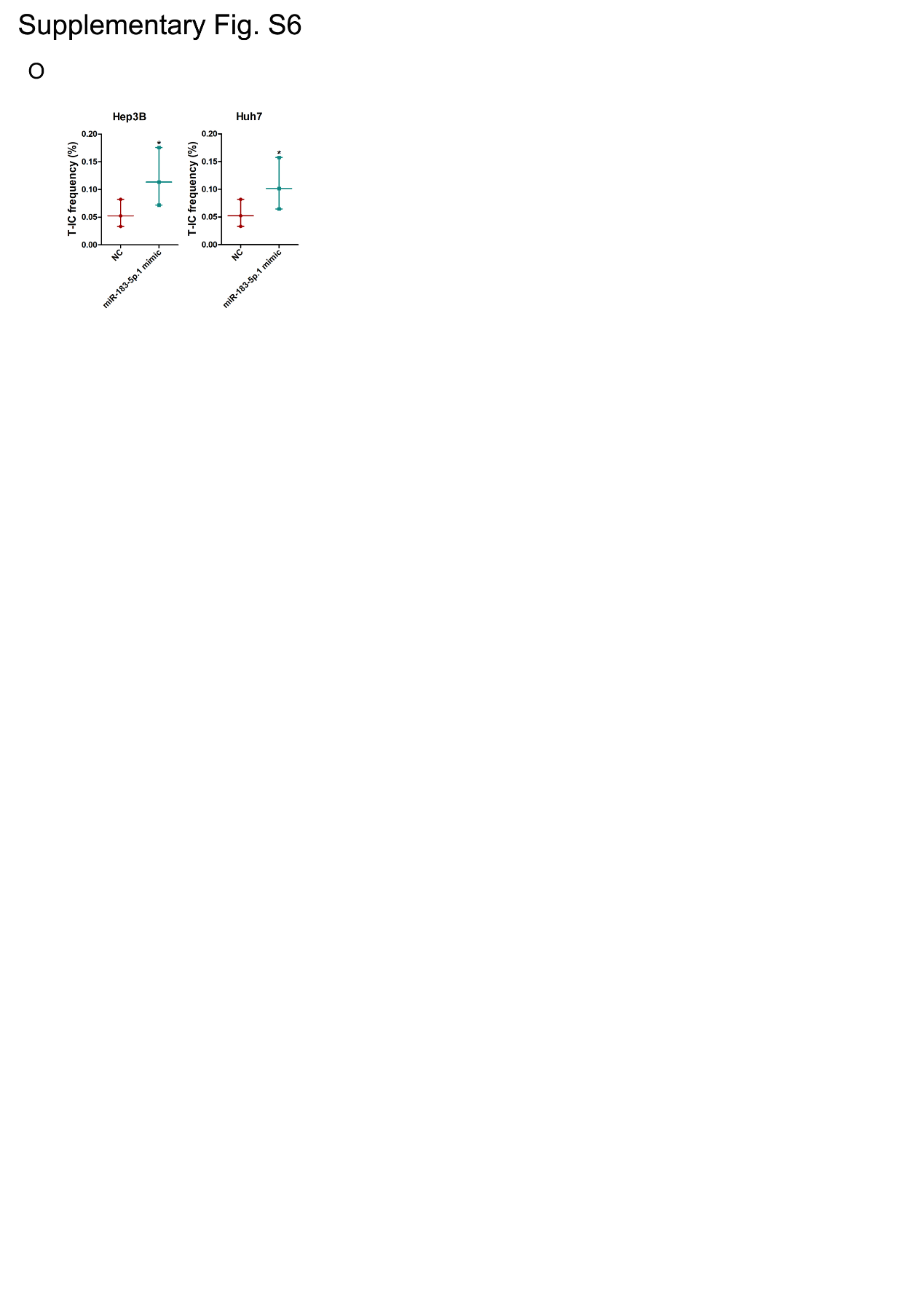


**Supplementary Fig. S6**

**A.** The correlation between the level of MUC15 and miR-183-5p.1 in HCC tumoral samples (n=40) was determined by real-time PCR analysis.

**B.** The relative expression of miR-183-5p.1 was determined in fresh RNA samples of normal liver (n=40) and HCC tissue (n=40).

**C.** Representative staining for miR-183-5p.1.

**D.** Real-time PCR analysis miR-183-5P.1 expression in sorted EpCAM^+^ or CD24^+^ primary HCC cells relative to negative cells.

**E.** Real-time PCR analysis of miR-183-5P.1 expression in primary HCC adherent cells and spheres.

**F.** Real-time PCR analysis of miR-183-5P.1 expression in in serial passages of primary HCC spheroids.

**G.** Real-time PCR analysis of miR-183-5P.1 expression in primary HCC adherent, spheres and re-adherent cells.

**H.** Real-time PCR analysis miR-183-5P.1 expression in sorted EpCAM^+^ or CD24^+^ HCC cells relative to negative cells.

**I.** Real-time PCR analysis of miR-183-5P.1 expression in HCC adherent cells and spheres.

**J.** Real-time PCR analysis of miR-183-5P.1 expression in in serial passages of HCC spheroids.

**K.** Real-time PCR analysis of miR-183-5P.1 expression in HCC adherent, spheres and re-adherent cells.

**L.** Hep3B/CSQT-2 cells infected with miR-183-5P.1 overexpression virus were subjected to real-time PCR analysis.

**M&N.** Real-time PCR analysis of liver T-IC markers or pluripotent transcription factors in spheroids generated from miR-183-5P.1 overexpression hepatoma cells and control cells.

**O**. The frequency of liver T-ICs in miR-183-5p.1 overexpression hepatoma cells and control cells were compared by *in vitro* limiting dilution assay.

**
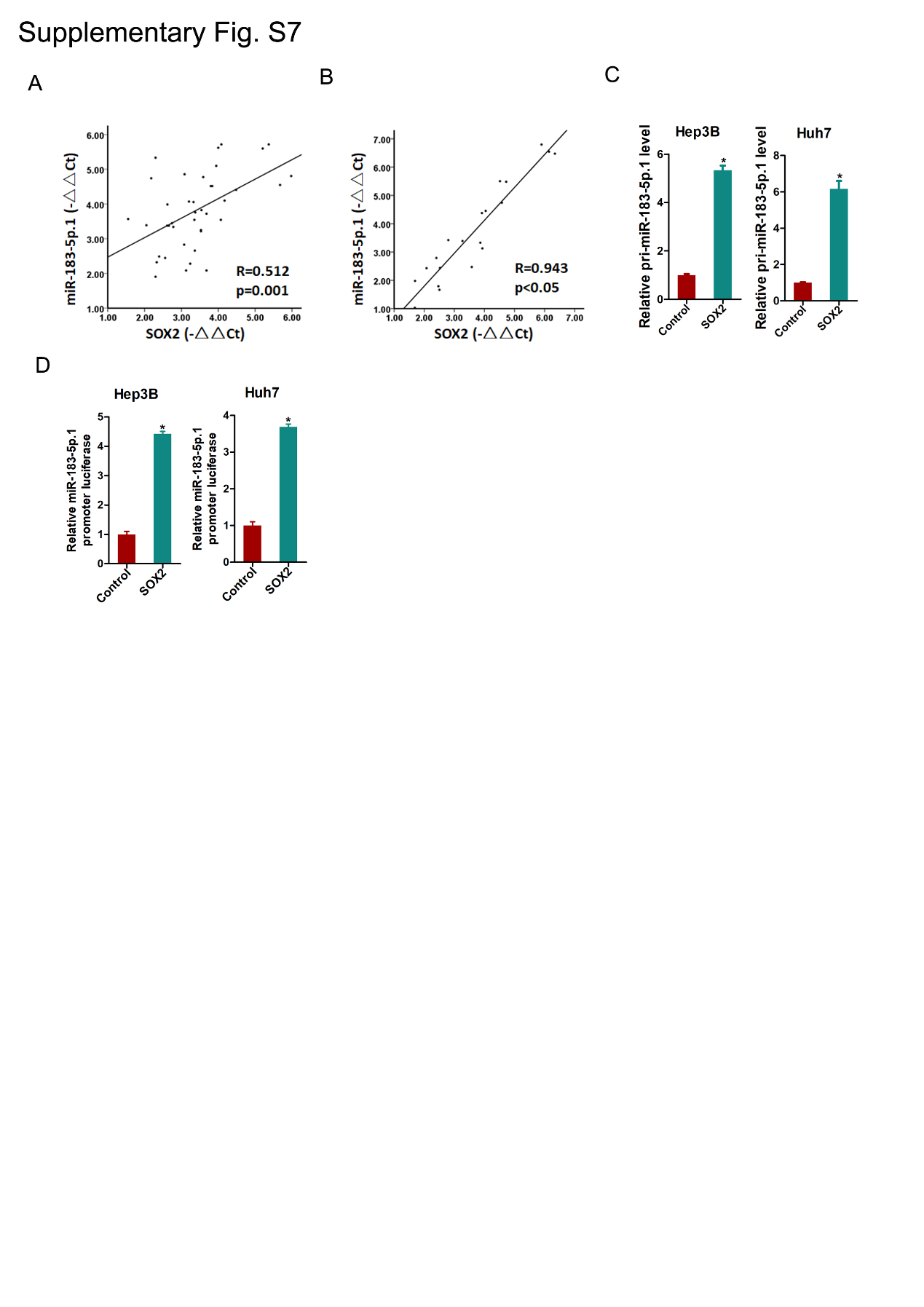
**

**Supplementary Fig. S7**

**A.** The correlation between the level of SOX2 and miR-183-5p.1 in HCC tumoral samples (n=40) was determined by real-time PCR analysis.

**B.** The correlation between the level of SOX2 and miR-183-5p.1 in EpCAM^+^ primary HCC cells (n=20) was determined by real-time PCR analysis.

**C.** Real-time PCR analysis of pri-miR-183-5p.1 in spheroids generated from SOX2 overexpression hepatoma cells and control cells.

**D.** The luciferase reporter activity of miR-183-5p.1 promoter was measured in SOX2 overexpression and control HCC spheres.

**
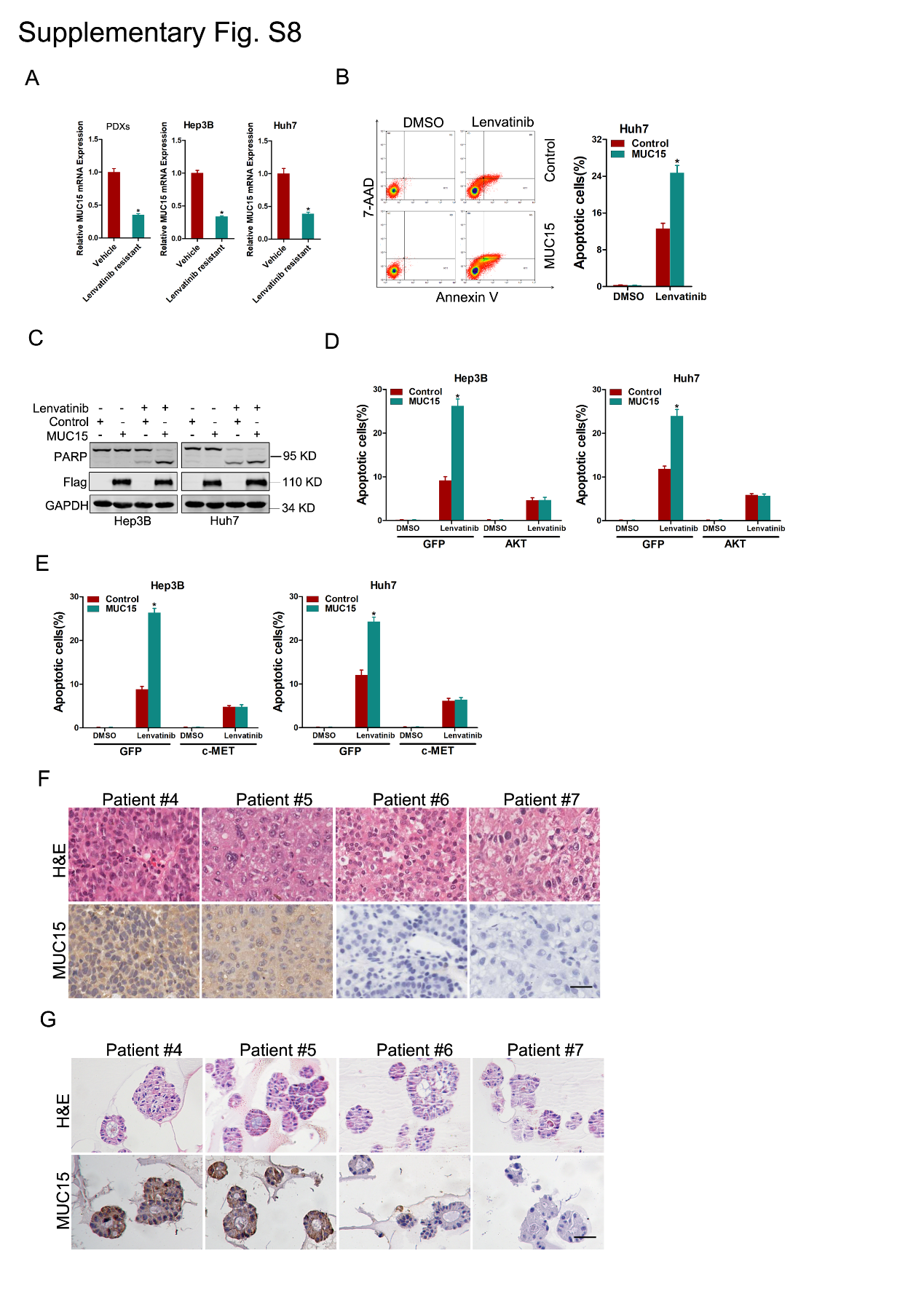
**

**Supplementary Fig. S8**

**A.** Real-time PCR analysis of MUC15 expression in lenvatinib-resistant HCC PDXs or cell lines.

**B.** Huh7 MUC15 and control cells were treated with lenvatinib (10 μM) for 48 hours and their apoptosis was examined by flow cytometry.

**C.** Hep3B/Huh7 MUC15 and control cells were treated with lenvatinib (10 μM) for 48 hours and their apoptosis was examined by western blot.

**D.** MUC15 overexpression cells and control hepatoma cells infected with AKT overexpression virus were treated with lenvatinib for 48 hours followed by cytometry analysis of apoptosis.

**E.** MUC15 overexpression cells and control hepatoma cells infected with c-MET overexpression virus were treated with lenvatinib for 48 hours followed by cytometry analysis of apoptosis.

**F.** H&E and IHC staining of MUC15 in the primary HCCs.

**G.** H&E and IHC staining of MUC15 in PDOs.

**Supplementary Tables**

**Supplementary Table 1. Clinicopathologic Features of 45 HCC Specimens in Cohort 1**

| Characteristics |  |  | MUC15 low  (n=23) | MUC15 high  (n=22) | p value |
| --- | --- | --- | --- | --- | --- |
| Age(year) | ≤50 |  | 8 | 5 | p>0.05 |
|  | >50 | | 15 | 17 |  |
| Gender | Male | | 18 | 19 | p>0.05 |
|  | Female | | 5 | 3 |  |
| HBsAg | Positive | | 18 | 18 | p>0.05 |
|  | Negetive | | 5 | 4 |  |
| AFP(μg/L) | ≤400 |  | 14 | 15 | p>0.05 |
|  | >400 |  | 9 | 7 |  |
| Tumor size(cm) | ≤5 | | 6 | 6 | p>0.05 |
|  | >5 | | 17 | 16 |  |
| Tumor number | Single |  | 20 | 19 | p>0.05 |
|  | Multiple |  | 3 | 3 |  |
| Portal vein tumor thrombus | Yes |  | 11 | 10 | p>0.05 |
|  | No |  | 12 | 12 |  |
| Encapsulation | Complete |  | 16 | 15 | p>0.05 |
|  | None |  | 7 | 7 |  |
| Pathologic satellite | Yes |  | 5 | 7 | p>0.05 |
|  | No |  | 18 | 15 |  |
| BCLC stage | A |  | 3 | 8 | p<0.05 |
|  | B or C |  | 20 | 14 |  |
| TNM | I-II |  | 3 | 4 | p>0.05 |
|  | III-IV |  | 20 | 18 |  |

HBsAg, hepatitis B virus surface antigen; AFP, α-fetoprotein; TNM, Tumor-Nodes-Metastasis; BCLC, Barcelona Clinic Liver Cancer Staging.

**Supplementary Table 2. Antibody List.**

| **Antigens** | **Manufacturer** | **Application** |
| --- | --- | --- |
| MUC15 | (ab224468) Abcam, USA | 1:1000 for WB or  1:50 for IHC |
| p-c-MET | (#3077) Cell Signaling Technology, Beverly, MA | 1:1000 for WB or  1:50 for IHC |
| c-MET | (#8198) Cell Signaling Technology, Beverly, MA | 1:1000 for WB or  1:50 for IHC |
| p-AKT | (66444-1-Ig) Proteintech Group, China | 1:500 for WB or  1:50 for IHC |
| AKT | (60203-2-Ig) Proteintech Group, China | 1:500 for WB |
| SOX2 | (ab92494) Abcam, Cambridge, MA | 1:1000 for WB |
| Flag | (ab205606) Abcam, Cambridge, MA | 1:1000 for WB or  1:50 for IP |
| HA | (ab9110) Abcam, Cambridge, MA | 1:1000 for WB |
| AFP | (14550-1-AP) Proteintech Group, China | 1:100 for IHC |
| CD133 | (ab222782) Abcam, Cambridge, MA | 1:50 for IHC |
| CD24 | (ab199140) Abcam, Cambridge, MA | 1:50 for IHC |
| CD90 | (ab92574) Abcam, Cambridge, MA | 1:50 for IHC |
| EpCAM | (#93790) Cell Signaling Technology, Beverly, MA | 1:50 for IHC |
| Ki67 | (27309-1-AP) Proteintech Group, China | 1:100 for IHC |
| β-actin | (CL594-60008) Proteintech Group, China | 1:2000 for WB |
| GAPDH | (60004-1-Ig) Proteintech Group, China | 1:5000 for WB |

**Supplementary Table 3. Primer List.**

| **Gene** | **Forward primer (5’-3’)** | **Reverse primer (5’-3’)** | |
| --- | --- | --- | --- |
| MUC15(Human) | Forward (5*′*- 3*′*) | CTTGCCACATCAAGGGATTGT | |
|  | Reverse (5*′*- 3*′*) | TGGCTCCCCGATAGAAGTGA | |
| β-actin(Human) | Forward (5*′*- 3*′*) | GGCCCAGAATGCAGTTCGCCTT | |
|  | Reverse (5*′*- 3*′*) | AATGGCACCCTGCTCACGCA | |
| MUC15(Mouse) | Forward (5*′*- 3*′*) | CCTGGGTGCTTCACTGCTTA | |
|  | Reverse (5*′*- 3*′*) | TGGGTTGTAGTAACTGGTTCGT | |
| β-actin(Mouse) | Forward (5*′*- 3*′*) | GTGACGTTGACATCCGTAAAGA | |
|  | Reverse (5*′*- 3*′*) | GCCGGACTCATCGTACTCC | |
| CD133(Mouse) | Forward (5*′*- 3*′*) | ACAGTTTAGTGGCACTCTCT | |
|  | Reverse (5*′*- 3*′*) | TAAATCTCAACATCAGGACA | |
| CD24(Mouse) | Forward (5*′*- 3*′*) | CTACCGTAAGTTTTTCCAAT | |
|  | Reverse (5*′*- 3*′*) | ACTACTGTACGGGTAAGCAT | |
| CD90(Mouse) | Forward (5*′*- 3*′*) | CCAAGTCCTTCAAATATCTC | |
|  | Reverse (5*′*- 3*′*) | CAAAATGACAGCTAGTGGTA | |
| EpCAM(Mouse) | Forward (5*′*- 3*′*) | ATCAAATTTTCCTAGCATGT | |
|  | Reverse (5*′*- 3*′*) | CCTCCCAAGTACAGAATTAC | |
| CD133(Human) | Forward (5*′*- 3*′*) | AGAGGAAGCCGCAAC | |
|  | Reverse (5*′*- 3*′*) | CTGGCTCGTGAATTATTTAT | |
| CD24(Human) | Forward (5*′*- 3*′*) | GCAAACAGATGTGTTCTTAAT | |
|  | Reverse (5*′*- 3*′*) | TCATCCCTAAGATCAAGTTT | |
| CD90(Human) | Forward (5*′*- 3*′*) | GAATACGGAAATGGATTAAG | |
|  | Reverse (5*′*- 3*′*) | GTATTCATTTCCTCTGGTCT | |
| EpCAM(Human) | Forward (5*′*- 3*′*) | | CGCAGCTCAGGAAGAATGTG |
|  | Reverse (5*′*- 3*′*) | | TGAAGTACACTGGCATTGACGA |
| SOX2(Human) | Forward (5*′*- 3*′*) | | TGGAGAAGGAATGGTCCACTTC |
|  | Reverse (5*′*- 3*′*) | | GGATAAGTACACGCTGCCCG |
| OCT4(Human) | Forward (5*′*- 3*′*) | | ATGTGCGCGTAACTGTCCAT |
|  | Reverse (5*′*- 3*′*) | | CTGCAGTGTGGGTTTCGGGCA |
| Nanog(Human) | Forward (5*′*- 3*′*) | | AATACCTCAGCCTCCAGCAGATG |
|  | Reverse (5*′*- 3*′*) | | TGCGTCACACCATTGCTATTCTTC |
| c-Myc(Human) | Forward (5*′*- 3*′*) | | CCCTCCACTCGGAAGGACTA |
|  | Reverse (5*′*- 3*′*) | | GCTGGTGCATTTTCGGTTGT |
| miR-183-5p.1(Human) | Forward (5*′*- 3*′*) | | TATGGCACTGGT AGAATTCACT |
|  | Reverse (5*′*- 3*′*) | | GCGAGCACAGAATTAATACGAC |
| U6(Human) | Forward (5*′*- 3*′*) | | CTCGCTTCGGCAGCACA |
|  | Reverse (5*′*- 3*′*) | | AACGCTTCACGAATTTGCGT |
| sh-MUC15 | 5’- GGAAUUCUAGCUACUACAATT -3’ | | |
| si-c-MET | 5’- ACAAGAUCGUCAACAAAAA -3’ | | |
| si-SOX2 | 5’-CCAUGGAUUUAUUCCUAAATT-3’ | | |
