# Supplementary figures and images for: Downregulation of MUC15 by miR-183-5p.1 promotes liver tumor-initiating cells properties and tumorigenesis via regulating c-MET/PI3K/AKT/SOX2 axis
Source: Cell Death Dis. 2022 Mar 2;13(3):200. doi: 10.1038/s41419-022-04652-9 (PMC8891362; doi:10.1038/s41419-022-04652-9)

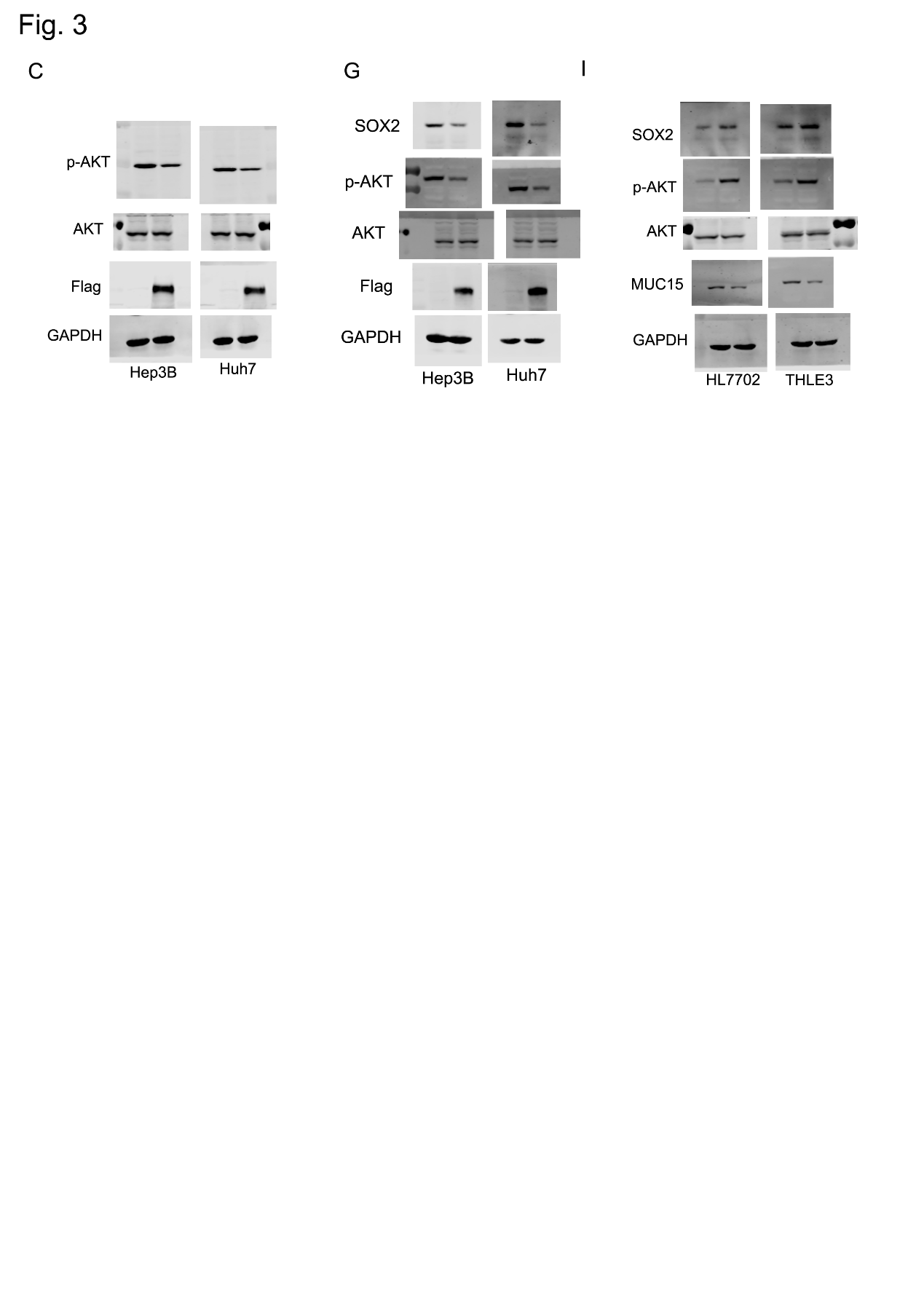


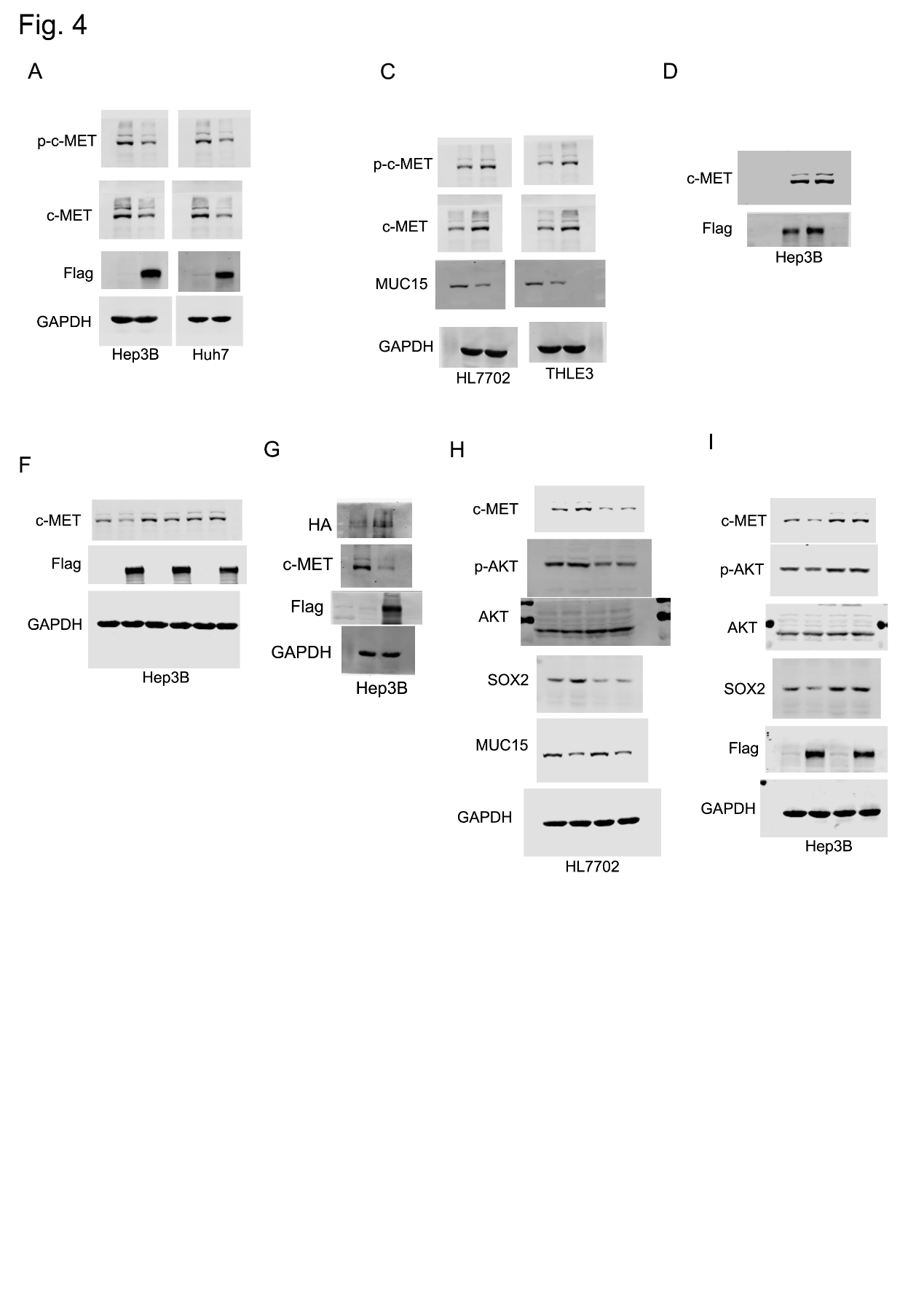


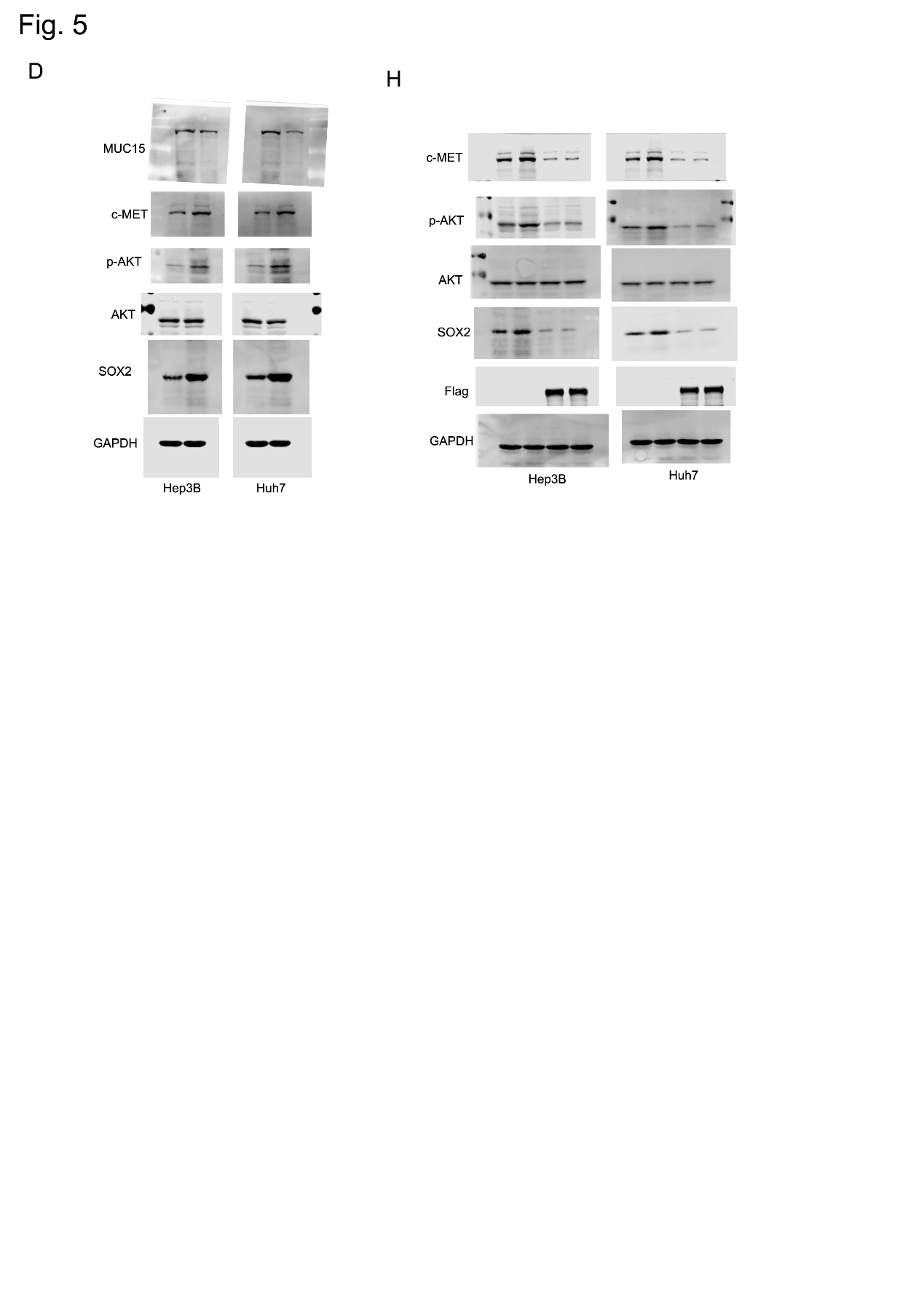


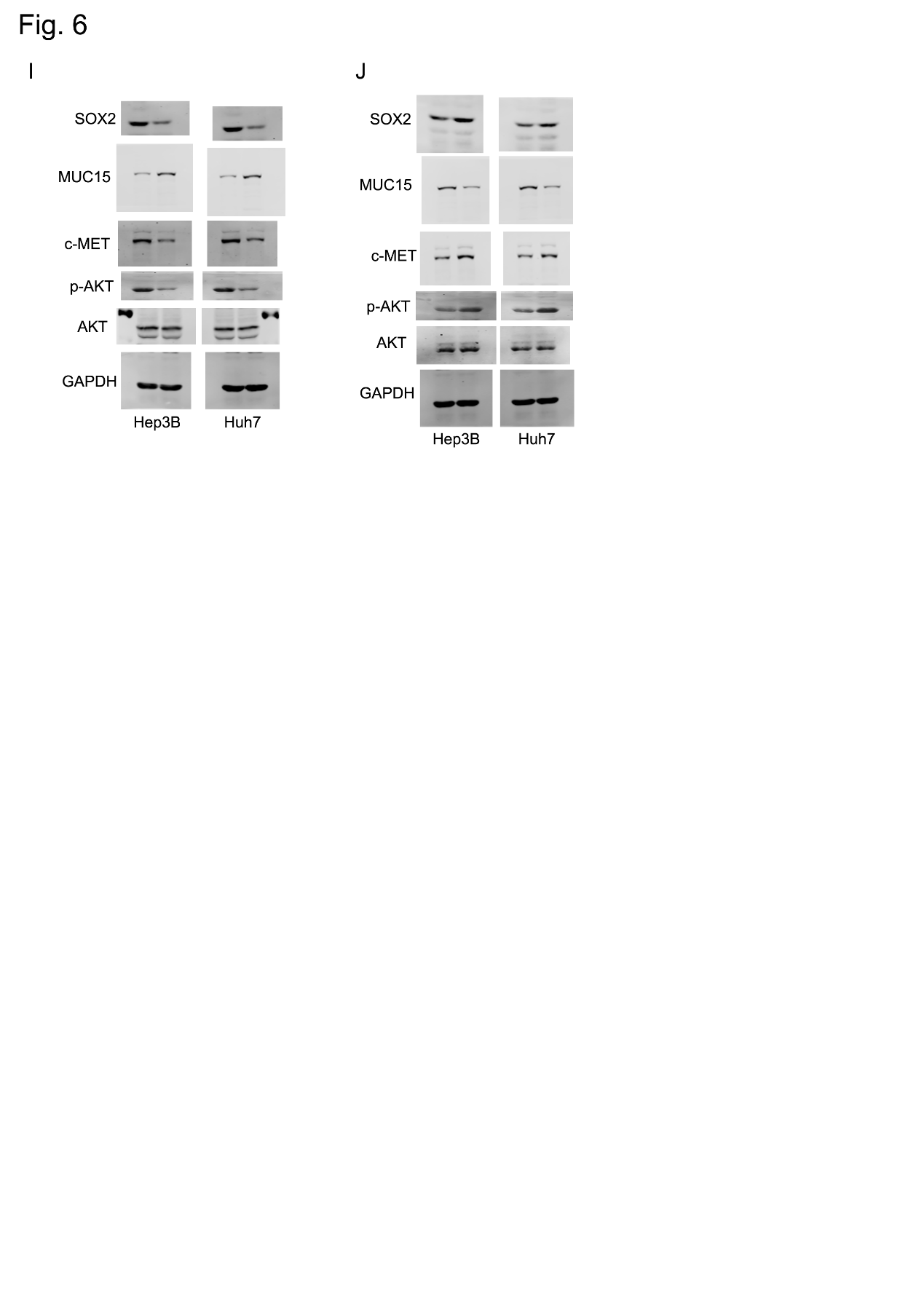


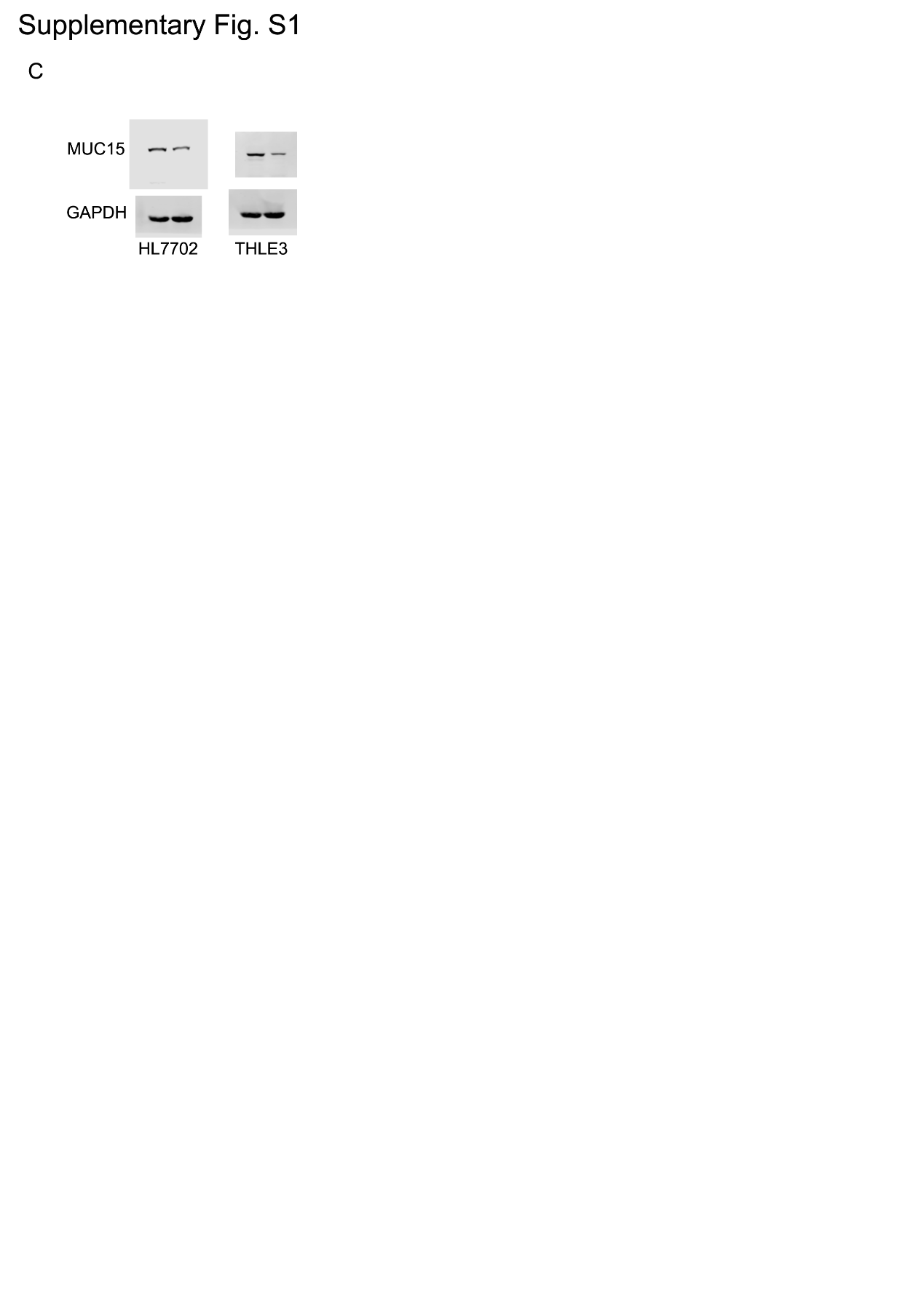


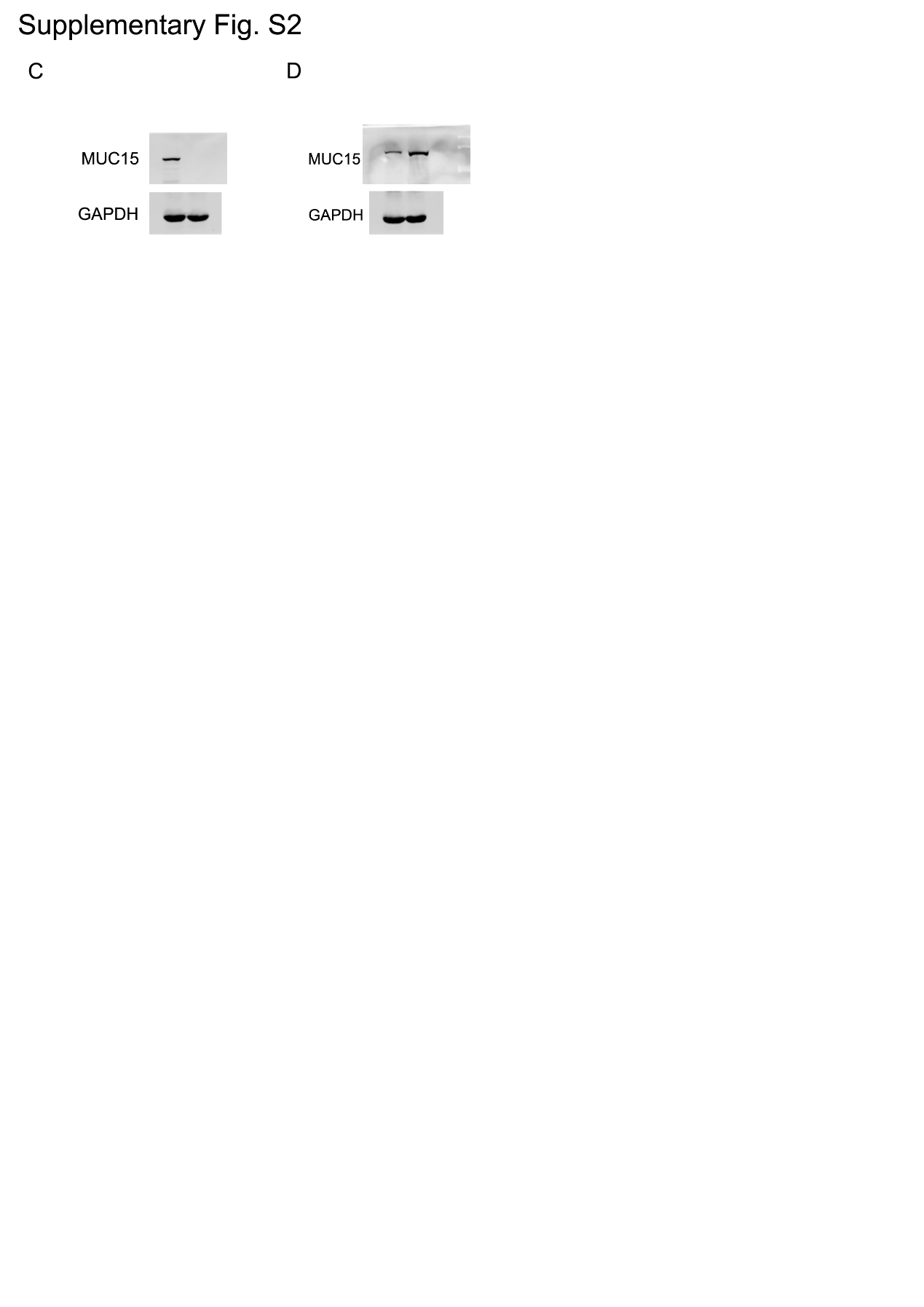


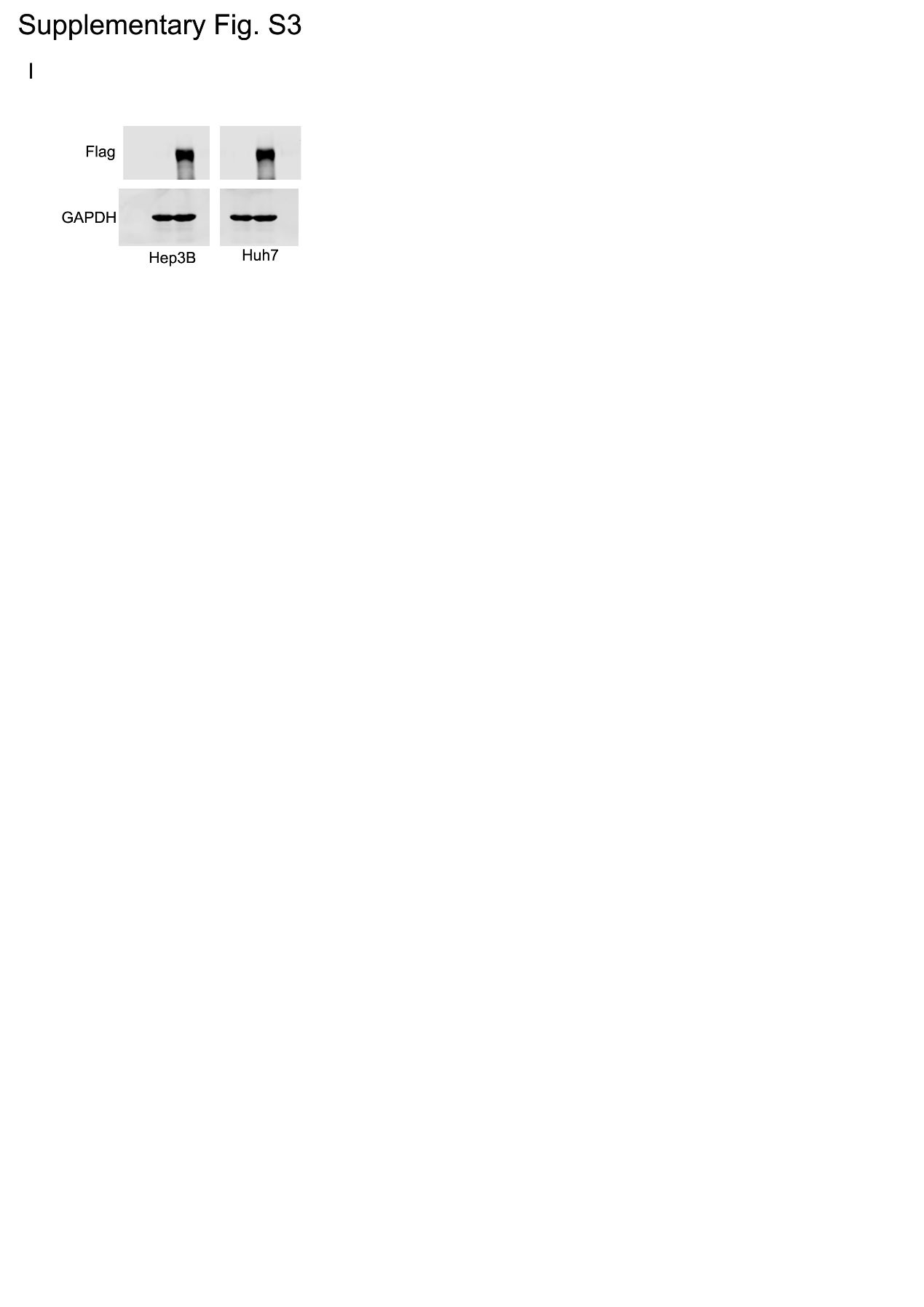


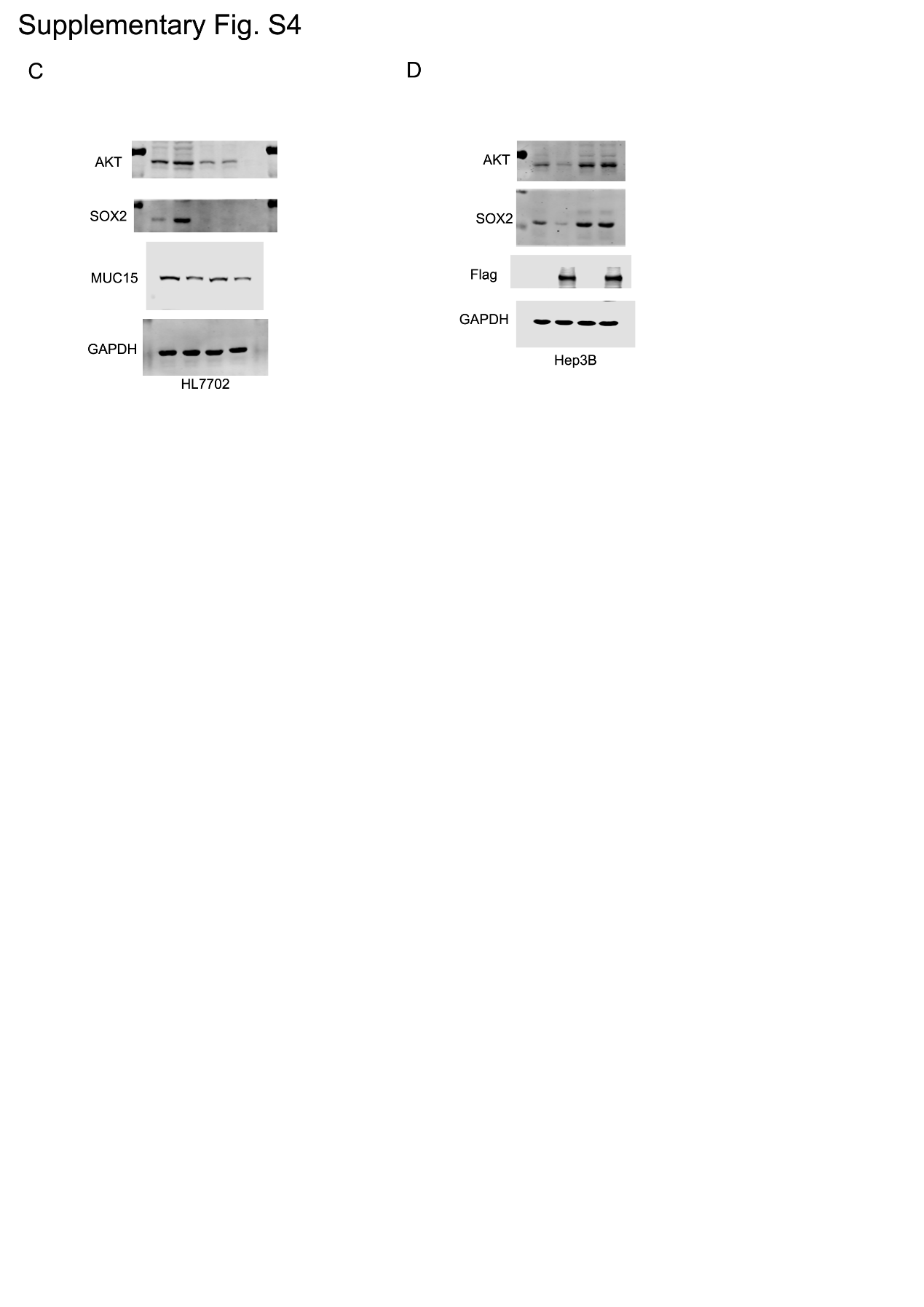


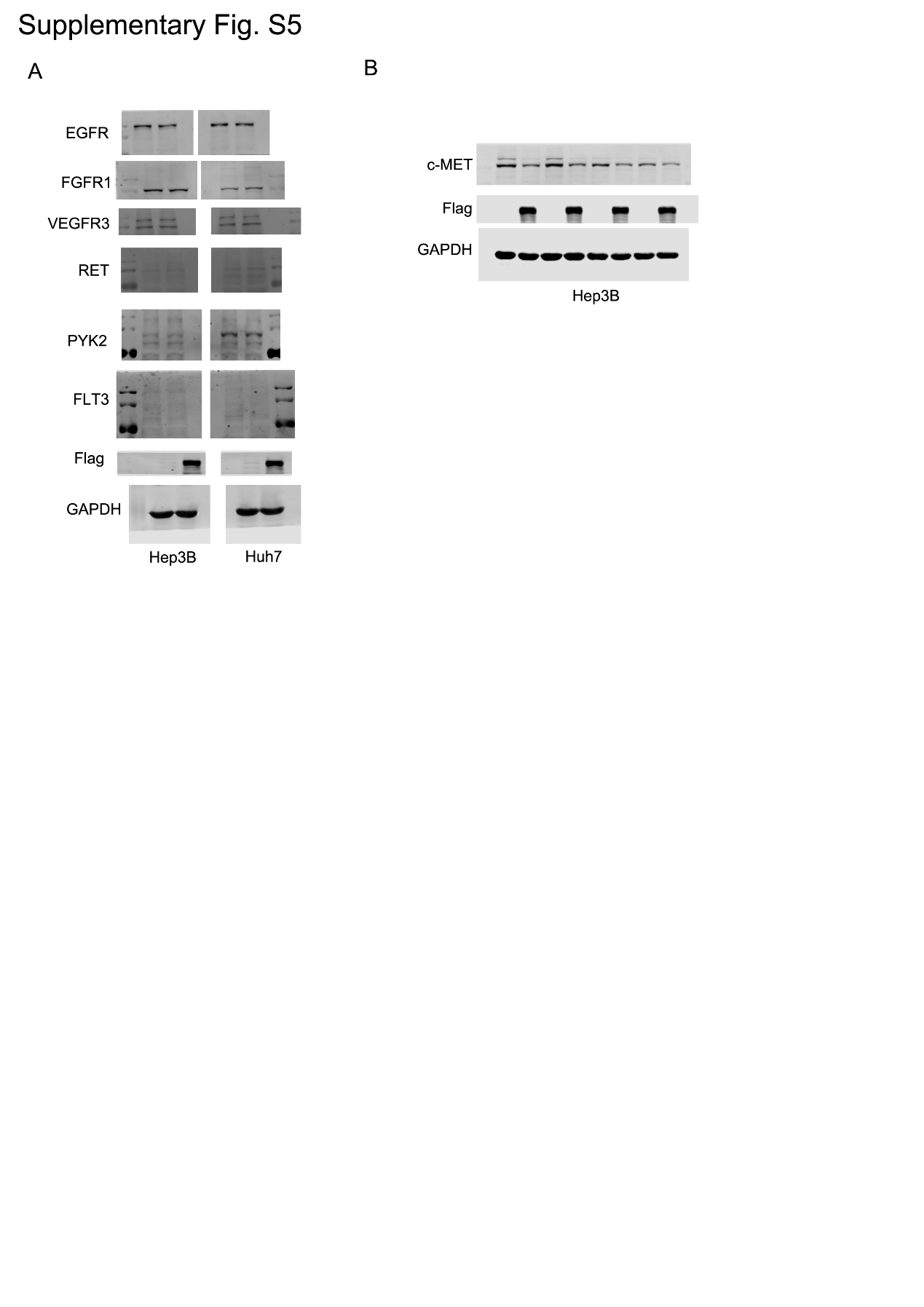


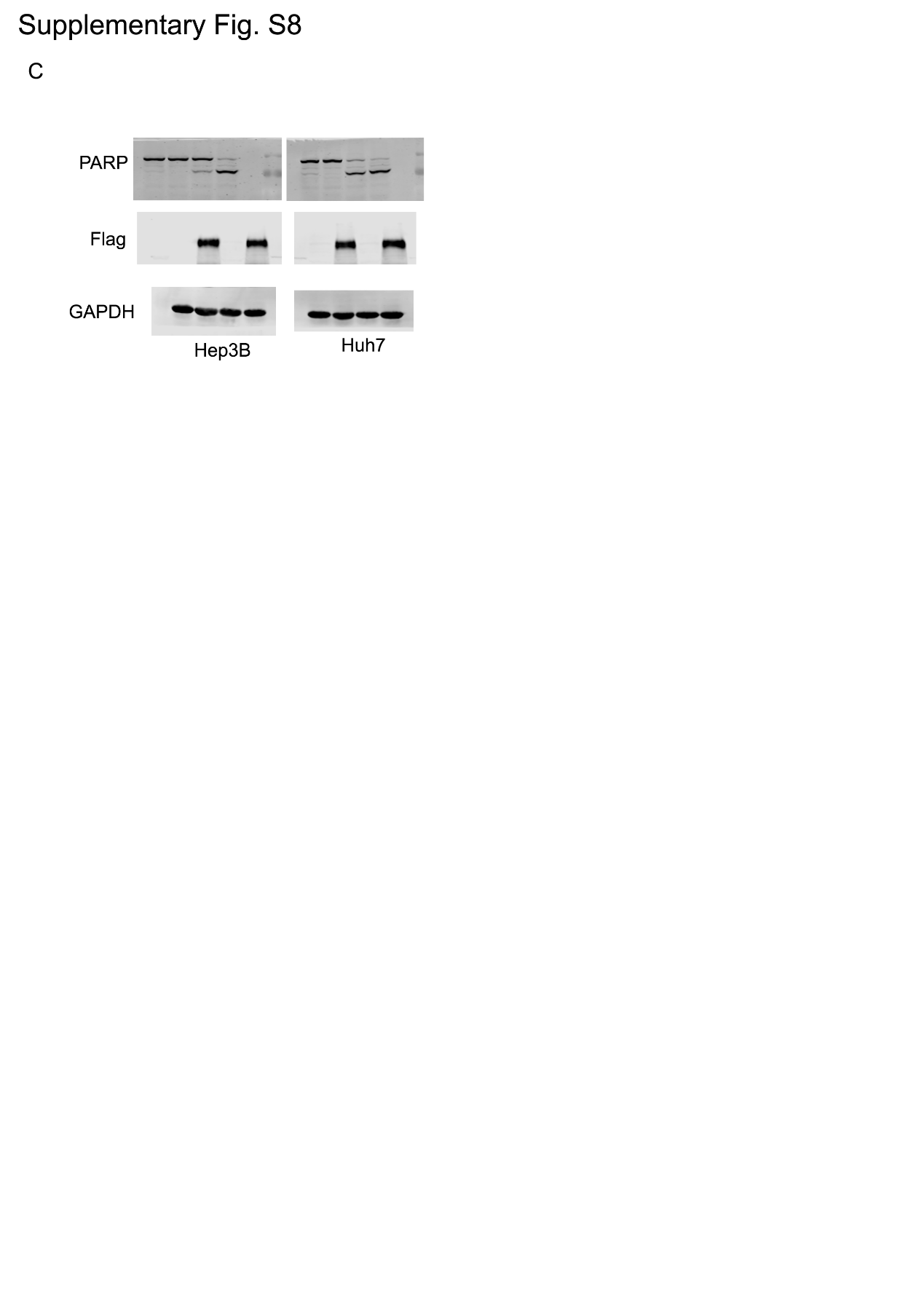

Supplement: Supplementary file 2 — original western blots [file 41419_2022_4652_MOESM2_ESM.docx]
